# Effects of motor and cognitive‐motor training on cognitive performance in healthy older adults

**DOI:** 10.1111/jnp.70043

**Published:** 2026-03-20

**Authors:** Silvia Gobbo, Elisa Galli, Daniele Romano

**Affiliations:** ^1^ Department of Psychology University of Milano‐Bicocca Milan Italy; ^2^ Mind and Behavior Technological Center University of Milano‐Bicocca Milan Italy

**Keywords:** aging, cognitive functions, cognitive training, motor training, well‐being

## Abstract

With a rapidly aging global population, identifying effective strategies to preserve cognitive health and functional independence is increasingly important. This study investigated the effects of motor and combined cognitive‐motor training on cognitive performance and well‐being in healthy older adults against a control condition. Participants completed four assessments, each spaced 1 month apart, which served as the control condition. The first two assessments constituted a double‐baseline period during which no training was administered. The third and fourth assessments followed the motor and cognitive–motor training phases, respectively, with the order of training counterbalanced across participants. Training was delivered to 23 individuals at home via the rehability telerehabilitation platform, which provided daily serious games targeting motor and cognitive skills. Outcomes were assessed using standardized cognitive tests and validated well‐being questionnaires. Sample size was determined a priori assessing the risk of misconclusion, and Bayesian statistics were used to obtain nuanced yet robust results. The study was preregistered. Results indicated that both motor and cognitive‐motor training led to improvements in key cognitive domains, including memory (BF_10_ = 156.837), processing speed (BF_10_ = 4.687) and inhibitory control (BF_10_ = 101.559). These gains were observed relative to the control condition, suggesting a key role of the interventions rather than a mere effect of time, test learning or spontaneous improvement. No significant changes were observed in self‐reported mood or overall well‐being suggesting that cognitive and psychological outcomes might be impacted differently by the training.

## INTRODUCTION

Population aging is a global phenomenon. In 2020, people aged 65 and over accounted for about 10% of the global population and are expected to more than double by 2050 (Tragaki, 2024). In Italy, taken as a concrete example as it is the context where this study was carried out, as of 1 January 2024, adults aged 65 or older make up 24.7% of the population (Istituto Nazionale di Statistica [ISTAT], 2025). This demographic shift carries significant implications: although life expectancy has increased, research suggests that the proportion of life spent in good health has remained relatively stable. As a result, these additional years are often marked by reduced well‐being, including both physical and cognitive decline (Gavelin et al., 2020).

A negative consequence of aging is the increased risk of Mild Cognitive Impairment (MCI), a condition that affects between 3% and 19% of older adults. While MCI does not qualify as dementia, a significant proportion—34%—of MCI cases progress to dementia within a few years, with 28% specifically developing Alzheimer's disease (Hu et al., 2017). This progression not only impacts individuals but also places a substantial burden on caregivers and society at large (Javaid et al., 2021). However, lifestyle factors may play a protective role against the onset of cognitive impairment, including both MCI and dementia, and such preventive strategies can be implemented already during healthy aging. If increased life expectancy is accompanied by good health, aging may in this way offer opportunities for continued participation and contribution to society. Achieving this requires effective interventions based on primary and secondary prevention strategies. In this regard, maintaining an active lifestyle is essential for promoting healthy aging and mitigating age‐related challenges (Giaquinto et al., 2023).

A key factor influencing cognitive functioning during aging is cognitive reserve (CR), a multifaceted construct shaped by individual attributes and lifelong experiences, such as education, occupation, leisure activities and social engagement (Stern, 2009). CR acts as a protective buffer against dementia, with activities that enhance it—even early in life—showing potential to support cognitive well‐being later in life (Wu et al., 2016). Moreover, CR enhances mental health by fostering resilience to stress and cognitive decline, even before aging sets in (Porricelli et al., 2024). More recently, the concept of motor reserve has been introduced, referring to the physical activity (PA) engaged in throughout life that helps compensate for age‐related motor and cognitive decline (Pucci et al., 2024).

Leveraging on the fact that both cognitive and motor reserves are modifiable and protective against pathological aging, various training programmes have been developed to boost cognitive function and well‐being in older adults (Oberlin et al., 2022).

When it comes to cognitive training, several meta‐analyses of randomized controlled trials (RCTs) have demonstrated positive effects on key cognitive functions in healthy older adults. Pooled effect size estimates indicate small to moderate improvements in attention, processing speed, executive functions, visuospatial abilities and memory (Basak et al., 2020; Chiu et al., 2017; Tetlow & Edwards, 2017). However, effect sizes can vary considerably, likely due to differences in training protocols and study designs. Importantly, baseline repeated evaluations are not always included in cognitive assessments, making it difficult to distinguish between spontaneous improvements and the effects of the intervention. Importantly, preregistration is rarely adopted, limiting the transparency and generalizability of findings. Furthermore, active control training is not always implemented, raising concerns that reported positive effects may result from comparisons with passive control groups—where participants receive no intervention and have no interaction with researchers between the pre‐test and post‐test phases (Tsai et al., 2018).

PA also proved to be effective for supporting cognitive function in older adults. Engaging in structured PA programmes has been shown to enhance overall cognition in individuals without cognitive impairment and positively influence cognitive domains that are typically affected by aging (Erickson et al., 2019; Stillman et al., 2020). A meta‐analysis of 39 RCTs found modest improvements in global cognition, with specific gains in attention, memory and executive function (Northey et al., 2018).

Notably, combining motor and cognitive‐motor training may be more effective than single‐modality interventions in enhancing cognitive function. Accumulating evidence suggests that integrated training approaches offer both additive and potentially synergistic effects, resulting in greater cognitive gains (Sipilä et al., 2021; Ten Brinke et al., 2020).

Furthermore, while cognitive performance is frequently assessed, subjective well‐being remains an underexplored outcome, despite its importance for overall quality of life in aging (Bureš et al., 2016; Delle Fave et al., 2018).

As an alternative to standardized cognitive training, which has been shown to be effective in improving cognitive outcomes (Pegoraro et al., 2026), serious games—defined as games developed for purposes beyond entertainment, such as education, training or rehabilitation—have gained increasing attention (Damaševičius et al., 2023). Among these, exergames, which combine physical exercise with interactive gameplay, have emerged as an engaging approach to promote both physical and cognitive health. Exergaming integrates motor activity with cognitively demanding tasks (Moret et al., 2024) and has been proposed as a promising intervention for active aging (Moret et al., 2022; Yang et al., 2023). Despite growing interest in cognitive and physical training for older adults, existing studies often lack methodological rigour, including preregistration and appropriate use of control conditions, which limits generalizability. Moreover, few studies directly compare the cognitive effects of motor versus cognitive‐motor training within the same participants, and even fewer assess broader outcomes like subjective well‐being. Eventually, as many recent studies incorporate new technologies and methods, it is essential to consider participant compliance—namely, their level of engagement with the programme—as well as their user experience with the tools employed.

The present study aimed to evaluate whether motor training and combined cognitive‐motor training, delivered through serious games can enhance cognitive performance and well‐being in healthy older adults. A longstanding issue in the literature concerns the observation that cognitive training often leads to so‐called near transfer—namely, improvements closely related to the trained tasks—rather than far transfer, which refers to gains in more general cognitive abilities (Barnett & Ceci, 2002; Sala et al., 2019). For this reason, the cognitive training component was designed to include games that closely matched the cognitive functions assessed by the neuropsychological tests. At the same time, there is evidence that PA can exert broader, nonspecific benefits on cognition extending transfer (Bherer, 2015). Based on that, we hypothesized that motor training in combination with cognitive training would lead to even further improvements in cognitive functioning. Thus, we expected both interventions to positively impact cognition, with greater effects for the combined training.

To strengthen the methodological validity—often limited in previous research—we preregistered our hypotheses and analysis plan (https://aspredicted.org/s73s‐bz8k.pdf) and conducted a BFDA (Schönbrodt & Wagenmakers, 2018) a priori to determine the adequacy of the sample size. We employed a cross‐over design that included a control condition, allowing each participant to serve as their own control. This approach enhanced experimental sensitivity while retaining the inclusion of control conditions within the study. Furthermore, the study comprehensively assessed a broad range of cognitive domains and subjective health outcomes while accounting for key moderating factors such as cognitive and motor reserves, participant compliance, and user experience.

## METHODS

### Participants

A total of 24 participants were initially recruited for the study (M_age_ = 70.81, SD_age_ = 3.98; 17 females; Meducation = 13.43 years, SDeducation = 6.19). However, one participant dropped out after recruitment and could not be replaced, resulting in a final sample of 23 participants. The sample size was limited by logistical constraints, as the software used for training was only available for 9 months and we had access to a maximum of 10 home‐kits at a time. Given these limitations, we were able to organize at most two waves of data collection. Given these constraints, we investigated a priori the risks of incorrect conclusions associated with our design. We adopted a BFDA (Schönbrodt & Wagenmakers, 2018), the Bayesian equivalent of a power analysis. Assuming a medium effect size (*d* = .5) and a standard threshold for evidence (BF = 3), our sample size yields a low probability of misleading evidence in the case of true H0 (1.8%). This metric quantifies the risk that the Bayes Factor (BF) would provide support for the incorrect hypothesis and is conceptually similar—but not directly equivalent—to a frequentist α‐level.

Participants were recruited through retirement and aging associations. Each individual received a detailed explanation of the procedure and was asked whether they were interested in participating. Eligibility criteria included being over 65 years old, retired, and having both a Wi‐Fi connection and a television with an HDMI port at home to support installation of the training programme. Exclusion criteria included reporting neurological or psychiatric disturbances, and having physical disturbances that would prevent participants to do mild motor activity required for the training. To ensure compliance with these criteria, all participants were assessed during the first session using the MoCA (both as a baseline cognitive assessment and to detect potential cognitive impairment). In addition, a brief anamnestic interview was conducted to confirm that participants did not report any cognitive or physical conditions that might interfere with the training.

The study was deemed a minimal risk study by the Commissione per la Ricerca In Psicologia‐ CRIP (RM‐2024‐796) of University of Milano‐Bicocca, which works as an evaluation body under the mandate of the University Ethics Committee, to which it refers. All participants provided written informed consent in accordance with the Declaration of Helsinki. Data collection took place in two waves, beginning in April 2024 and concluding in November 2024.

### Materials

During the first session, both Cognitive and Motor Reserve were assessed using the Cognitive Reserve Index Questionnaire (Nucci et al., 2012) and the Motor Reserve Index Questionnaire (Pucci et al., 2024), respectively.

Materials used for repeated assessments included the following:

- The Montreal Cognitive Assessment (MoCA) was used to evaluate global cognitive functioning. We adopted the Italian standardization of the MoCA (Conti et al., 2015) along with two standardized parallel versions (Siciliano et al., 2019). Since there were four total assessment sessions, the original version of the MoCA was reused in the final session. The order of MoCA versions was counterbalanced across the two waves of data collection.
- The Rey Auditory Verbal Learning Test (RAVLT) was employed to assess learning and memory. We used the Italian standardization (Carlesimo et al., 1996) and two parallel word lists (Carlesimo et al., 2014). As with the MoCA, the original RAVLT version was used again in the fourth session.
- The Stroop Test was used to assess inhibitory control, using the Italian standardization (Caffarra et al., 2002). No parallel versions were employed, as the Stroop Test is not expected to involve a memory component, thereby minimizing learning effects.
- The Symbol Digit Modalities Test (SDMT) was used to assess speed processing, using the Italian standardization (Nocentini et al., 2006). No parallel versions were employed, as the SDMT is not expected to involve a memory component, thereby minimizing learning effects.
- The Trail Making Test (TMT), versions A and B, was administered to evaluate selective and alternating attention. The Italian standardization was used (Giovagnoli et al., 1996). No parallel versions were used due to the minimal memory involvement in TMT.
- The 36‐Item Short Form Health Survey (SF‐36, Apolone & Mosconi, 1998) was administered to assess general well‐being.
- The Positive and Negative Affect Schedule (PANAS, Terracciano et al., 2003) was used to evaluate mood.
- Following the completion of each training programme, participants also completed the User Experience Questionnaire (UEQ; Laugwitz et al., 2008).

### Serious games

The digital solution used in this study was Rehability (Rehability, Imaginary srl: https://www.rehability.me/#gallery), a platform featuring a variety of serious games designed for neurological rehabilitation. The platform includes both motor and combined cognitive‐motor training exercises. All games are operated using an RGBD camera (Orbbec3D Astra PRO), which tracks participants' movements through a Nuitrack software (www.nuitrack.com). After an initial calibration, participants control the games through body movements.

Each participant received a home kit, which included an RGBD camera, a mini‐computer (Lattepanda 2 or Lattepanda 3 delta) with pre‐installed software, and an HDMI cable to connect the mini‐computer to a television.

We developed two separate training programmes using Rehability: a motor training programme and a cognitive–motor training programme. Because all exercises were pre‐existing and provided by the platform, the intervention design involved selecting and combining available exercises rather than creating novel tasks.

The cognitive–motor training programme consisted of exercises explicitly designed to engage specific cognitive functions, including selective, divided, and alternating attention, categorization processes, and cognitive flexibility. Exercises were selected to maximize overlap with the cognitive domains assessed by the neuropsychological battery (e.g., Stroop, SDMT, TMT, RAVLT), thereby allowing the investigation of near‐transfer effects. In addition to cognitive exercises, cognitive–motor programme included also purely motor tasks. Exercises were grouped into sets of comparable tasks and rotated across consecutive days to ensure variability and to avoid daily repetition. Examples include Day and Night (response inhibition and rule switching), Rockets and Meteorites (selective attention and dual‐task processing) and Find It (visual search and categorization).

Conversely, the motor training programme was designed to minimize explicit cognitive demands and primarily involved whole‐body movements. This condition was intended to assess whether participation in structured PA alone could produce changes in cognitive performance via nonspecific or generalized mechanisms. Representative exercises included Jumping Jack, Skipass and Hot Air Balloon, which primarily required gross motor coordination, balance and postural control.

Each training session lasted approximately 20 min, and participants were allowed to complete one session per day. Participants were instructed directly in their homes on the functioning of the platform and games by SG (Post‐Doc following the project) and EG (Master student doing her thesis on the project): the first session was completed with the experimenters. This was done to ensure a correct understanding of the exercises. Participants were instructed to complete at least three sessions per week for each training programme. Each training programme lasted 1 month. Data were collected in two waves: the first wave took place from April to July 2024, and the second wave ran from September to November 2024.

### Procedure

Participants completed four assessment sessions over the course of 3 months. The first assessment took place immediately after they consented to participate in the study. This was followed by a 1‐month waiting period, which served as a control condition to account for potential spontaneous improvements or test–retest learning effects prior to the training programmes.

Following the waiting period, participants underwent a second assessment, after which the Rehability programme was installed on their television. During this same session, they received instructions on how to use the platform.

Next, participants began their first training programme, which was either the motor training or the cognitive‐motor training, assigned in a counterbalanced order across participants. After 1 month of training, they completed the third assessment session, and the second training programme was introduced and explained.

Participants then completed the second training over the following month. At the end of this period, a final (fourth) assessment was conducted, and all materials were retrieved by the research team.

**FIGURE 1:**
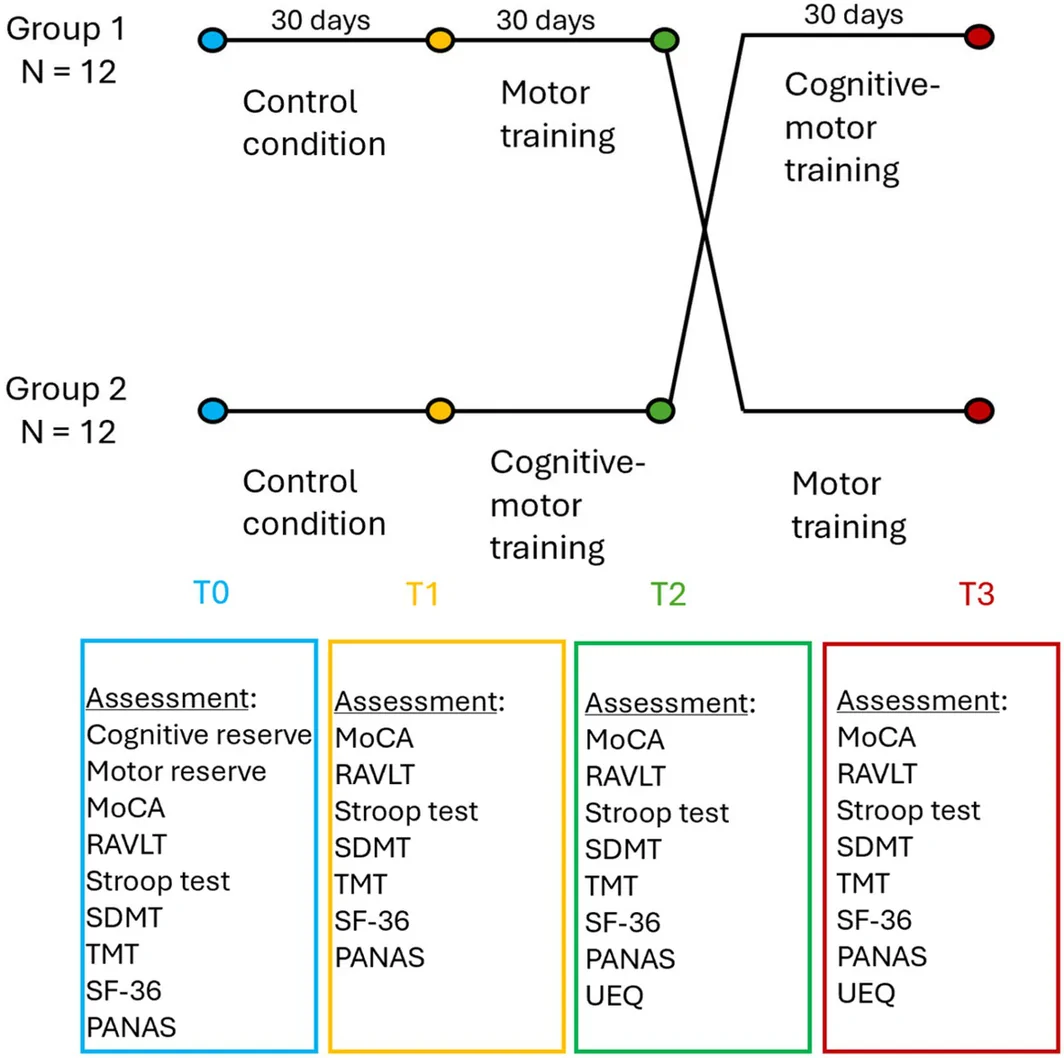
Graphical representation of the structure of the 3‐month experimental design. All participants took part in the study over a period of 3 months and completed four assessments, each spaced 1 month apart. T0 and T1 served as baseline assessments, while T2 and T3 were conducted following the cognitive‐motor and motor training interventions, administered in a counterbalanced order.

A visual representation of the experimental procedure is provided in Figure 1.

### Data analysis

Data were analysed using Bayesian statistics, a method that allows for evaluating evidence in favour of both the alternative and the null hypotheses. The primary output of this approach is the BF, which quantifies the extent to which the data support a hypothesis under opposing hypotheses. Specifically, BF_10_ represents the evidence of the data under the alternative hypothesis (H_1_) relative to the null hypothesis (H_0_). A BF of 1 indicates equal support for H_0_ and H_1_, while a BF_10_ greater than 1 favours H_1_ and a BF_10_ less than 1 favours H_0_. To interpret BF values, we followed traditional guidelines where a BF_10_ between 1 and 3 indicates anecdotal or inconclusive evidence in favour of H_1_, a BF of 3 or more is considered moderate evidence, and a BF above 10 constitutes strong evidence in favour of H_1_ (Jeffreys, 1961; Rouder et al., 2009).

### Pre‐registered analyses

We conducted the analyses as pre‐registered. Specifically, we calculated delta scores representing changes between consecutive timepoints: T1–T0 (baseline change), T2–T1 and T3–T2. Depending on the training sequence, T2–T1 and T3–T2 corresponded to either motor or cognitive training. Accordingly, we computed deltas relative to motor and cognitive training, regardless of their position in the sequence. These deltas were calculated for each outcome measure, including cognitive tests and well‐being assessments. Deltas were entered into a repeated measures Bayesian ANOVA (Rouder et al., 2012). Unlike the pre‐registered Bayesian *t*‐tests, this approach allowed us to include cognitive and motor reserve as covariates. Bayesian ANOVA is based on model comparison. First, we tested a null model that included cognitive and motor reserves as covariates, to control for potential confounding effects. We then tested an additional model incorporating the predictor training condition (i.e., baseline, cognitive training, motor training). For each model, we calculated the posterior probability (i.e., the probability of the model given the observed data). The most probable model after conditioning on the data was considered the best‐fitting model. BFs were then computed as the ratio of each model's posterior probability relative to that of the best model. We used the default prior settings for all analyses, that is a Cauchy prior with scale .5 for fixed effects, scale 1 for random effects and scale .354 for covariates. The model prior was set to uniform.

The ANOVA was followed by Bayesian post hoc tests to examine the effects of training in detail. Posterior odds were corrected for multiple comparisons by setting the prior probability of the null hypothesis to .5 across all contrasts, following the method proposed by Westfall et al. (1997). Because we then deviated from the pre‐registered plan, we reported the results of the pre‐registered analyses in the Supporting Information.

### Exploratory analyses

We conducted an exploratory analysis focused on examining the cumulative effect of the training interventions on the outcome measures, independently of the type of training.

To do so, a Bayesian ANOVA was conducted. Initially, a null model was tested, including cognitive and motor reserves as covariates to ensure the observed effects were not confounded by these individual differences. Additional models were tested that were: main effect of timepoint (T0, T1, T3), main effect of training order (Cognitive‐Motor or Motor‐Cognitive), the combination of the two main effects and the combination of the two plus their interaction. Note that T2 was excluded from the timepoint factor because the design already accounted for training type effects via the order variable. The ANOVA was followed by Bayesian post hoc tests to examine the effects of training in detail. Posterior odds were corrected for multiple comparisons as previously described.

We did not run a specific analysis on the UEQ, which we only looked at qualitatively through the spreadsheet provided by the authors. Values are converted in a scale from −3 (horribly bad) to +3 (extremely good) and values between −.8 and .8 represent a more or less neutral evaluation of the corresponding scale; values >.8 represent a positive evaluation and values <−.8 represent a negative evaluation (Laugwitz et al., 2008).

A summary Table 1 regarding the analysis plan and where to find details on the results is presented below.

**TABLE 1:** List of analyses, outcomes and where to find the details.

Analysis	Preregistered	Outcome	Where to find details of the result
Baseline vs. training deltas	Yes	No training‐type difference	Supporting Information
Motor vs. cognitive‐motor	Yes	Null	Supporting Information
Cumulative effect of training	No (exploratory)	Positive	Paper results

## RESULTS

### Pre‐registered analyses

Results of the pre‐registered analyses are available in the Supporting Information. A few tests showed a difference between baselines and other trainings, but no effects of training type emerged across any of the cognitive tests or well‐being domains assessed. Since no differences between training types were observed, we decided to collapse across the two interventions and focus on the effect of time, examining the cumulative effect of training—that is, the change from the baselines (T0 and T1) to the final assessment (T3)—in the exploratory analyses.

### Exploratory analyses

We then conducted an exploratory analysis to examine the cumulative effect of both training interventions. Descriptives for each measure and each timepoint are reported in Table 2 together with Cohen's *d*_*z*_ values. The results of this exploratory analysis are presented in Tables 3 and 4 and are coherent with the pre‐registered analyses. Interestingly, we found evidence of a cumulative effect of training on several cognitive measures (Table 3), although this effect did not extend to well‐being (Table 4). Specifically, improvements were observed in the RAVLT (both immediate and delayed recall), the Stroop Test (execution time) and the SDMT, when performance after training was compared with both the first and second baseline assessments. Full details of the results are provided in the Supporting Information.

**TABLE 2:** Descriptive values (i.e. mean and standard deviation) for each measurement in each timepoint together with Cohen's *d*_*z*_ values for the T3–T1 delta.

Test	T0 mean (SD)	T1 mean (SD)	T2 mean (SD)	T3 mean (SD)	Cohen's *d*_*z*_ for T3–T1 delta
MoCA	24.7 (2.75)	25.2 (2.50)	26.8 (1.59)	25.9 (1.91)	.33
RAVLTimm	45.9 (8.57)	41.3 (9.22)	43.5 (7.86)	50.3 (10.4)	.94
RAVLTdiff	9.91 (2.69)	8.57 (2.81)	9 (3.09)	10.8 (3.03)	.93
Stroop	23.2 (9.61)	21.5 (10.1)	17.1 (7.13)	17.1 (7.38)	.94
SDMT	42.2 (9.63)	43.1 (9.51)	45.5 (9.89)	47.1 (8.84)	.71
TMT‐A	46.2 (18.8)	45.3 (19.4)	41.9 (14.8)	39.9 (14.9)	.73
TMT‐B	110 (36.4)	113 (63.5)	101 (45.1)	90.6 (29)	.56
TMT‐BA	63.8 (30.7)	68.1 (51.7)	59 (39.2)	48.5 (22.6)	.47
PANAS_pos	35.2 (5.13)	35.9 (4.33)	36.5 (5.57)	33.6 (5.44)	.29
PANAS_neg	15.0 (5.45)	16.6 (5.27)	16.2 (6.82)	18.6 (6.83)	.39
SF‐36 physical functioning	0.87 (0.11)	0.83 (0.15)	0.82 (0.17)	0.82 (0.17)	.11
SF‐36 role of limitations due to physical health	0.59 (0.43)	0.67 (0.41)	0.73 (0.39)	0.65 (0.45)	.02
SF‐36 role of limitations due to emotional problems	0.73 (0.37)	0.81 (0.36)	0.89 (0.26)	0.79 (0.38)	.03
SF‐36 energy fatigue	0.62 (0.14)	0.63 (0.13)	0.63 (0.12)	0.58 (0.15)	.38
SF‐36 emotional wellbeing	0.80 (0.12)	0.78 (0.12)	0.76 (0.16)	0.72 (0.22)	.27
SF‐36 Social functioning	0.79 (0.19)	0.80 (0.21)	0.86 (0.18)	0.73 (0.27)	.34
SF‐36 Pain	0.67 (0.27)	0.72 (0.22)	0.74 (0.21)	0.64 (0.27)	.26
SF‐36 General Health	0.59 (0.15)	0.62 (0.15)	0.63 (0.13)	0.57 (0.15)	.40
SF‐36 Health change	0.44 (0.11)	0.46 (0.10)	0.54 (0.15)	0.44 (0.19)	.05

**TABLE 3:** Bayesian repeated‐measures ANOVA results for each cognitive outcome. For each test, the table reports Bayes factors (BF) quantifying the evidence for including the effects of Timepoint, Order and their Timepoint × Order interaction in the model. Inclusion Bayes factors compare models that include a given effect against otherwise identical models without that effect, thus expressing the relative evidence provided by the data for the presence of each factor (Rouder et al., 2012). BF inclusion >3 indicates moderate‐to‐strong evidence in favour of including the effect, whereas BF inclusion <1/3 indicates evidence against its inclusion. Post‐hoc comparisons are reported only when the evidence is conclusive (BF10 > 3 or BF10 < 1/3) and refer to pairwise comparisons between timepoints within the best‐supported model. All Bayes factors are expressed relative to the null hypothesis of no difference. Complete results are provided in the Supporting Information.

Test	BF timepoint	BF10 order	BF10 timepoint × order	Post‐hoc timepoint
Moca	3.844	0.437	0.282	T0 < T3 (BF_10_ = 4.716)
				T1 = T3 (BF_10_ = 0.311)
RAVLTimm	1756.292	0.420	0.333	T0 < T1 (BF_10_ = 5.371)
				T0 < T3 (BF_10_ = 16.934)
				T1 < T3 (BF_10_ = 156.837)
RAVLTdel	712.779	0.507	0.371	T0 < T1 (BF_10_ = 3.729)
				T0 < T3 (BF_10_ = 8.861)
				T1 < T3 (BF_10_ = 148.712)
STROOP	95.530	0.519	0.387	T0 = T1 (BF_10_ = 0.331)
				T0 < T3 (BF_10_ = 104.090)
				T1 < T3 (BF_10_ = 101.559)
SDMT	48.185	0.612	0.443	T0 = T1 (BF_10_ = 0.329)
				T0 < T3 (BF_10_ = 69.609)
				T1 < T3 (BF_10_ = 4.687)
TMT‐A	1.379	0.598	0.688	T0 = T1 (BF_10_ = 0.237)
				T0 = T3 (BF_10_ = 0.905)
				T1 < T3 (BF_10_ = 9.323)
TMT‐B	1.864	0.429	0.216	T0 = T1 (BF_10_ = 0.318)
				T0 < T3 (BF_10_ = 6.752)
TMT‐BA	0.542	0.361	0.138	T0 = T1 (BF_10_ = 0.308)
				T1 = T3 (BF_10_ = 0.692)

**TABLE 4:** Bayesian repeated‐measures ANOVA results for each cognitive outcome. For each test, the table reports Bayes factors (BF) quantifying the evidence for including the effects of Timepoint, Order and their Timepoint × Order interaction in the model. Inclusion Bayes factors compare models that include a given effect against otherwise identical models without that effect, thus expressing the relative evidence provided by the data for the presence of each factor (Rouder et al., 2012). BF inclusion >3 indicates moderate‐to‐strong evidence in favour of including the effect, whereas BF inclusion <1/3 indicates evidence against its inclusion. Post‐hoc comparisons are reported only when the evidence is conclusive (BF10 > 3 or BF10 < 1/3) and refer to pairwise comparisons between timepoints within the best‐supported model. All Bayes factors are expressed relative to the null hypothesis of no difference. Complete results are provided in the Supporting Information.

Test	BF inclusion timepoint	BF10 inclusion order	BF10 inclusion interaction	Post‐hoc
PANAS positive	0.267	0.270	0.078	T0 = T1 (BF_10_ = 0.260)
				T0 = T3 (BF_10_ = 0.326)
				T1 = T3 (BF_10_ = 0.704)
PANAS negative	1.096	0.459	0.295	T0 = T1 (BF_10_ = 0.353)
SF‐36 physical functioning	0.192	0.881	0.111	T0 = T1 (BF_10_ = 0.385)
				T0 = T3 (BF_10_ = 0.461)
				T1 = T3 (BF_10_ = 0.252)
SF‐36 role of limitations due to physical health	0.176	0.628	0.266	T0 = T1 (BF_10_ = 0.395)
				T0 = T3 (BF_10_ = 0.379)
				T1 = T3 (BF_10_ = 0.234)
SF‐36 role of limitations due to emotional problems	0.125	0.452	0.161	T0 = T1 (BF_10_ = 0.263)
				T0 = T3 (BF_10_ = 0.259)
				T1 = T3 (BF_10_ = 0.234)
SF‐36 emotional wellbeing	0.903	0.709	0.335	T0 = T1 (BF_10_ = 0.310)
				T1 = T3 (BF_10_ = 0.620)
SF‐36 energy fatigue	0.337	0.447	0.128	T0 = T1 (BF_10_ = 0.233)
				T0 = T3 (BF_10_ = 0.720)
				T1 = T3 (BF_10_ = 0.581)
SF‐36 Social functioning	0.225	0.529	0.221	T0 = T1 (BF_10_ = 0.257)
				T0 = T3 (BF_10_ = 0.314)
				T1 = T3 (BF_10_ = 0.496)
SF‐36 Pain	0.194	0.606	0.083	T0 = T1 (BF_10_ = 0.415)
				T0 = T3 (BF_10_ = 0.232)
				T1 = T3 (BF_10_ = 0.402)
SF‐36 General Health	0.396	0.379	0.118	T0 = T1 (BF_10_ = 0.797)
				T0 = T3 (BF_10_ = 0.233)
				T1 = T3 (BF_10_ = 0.870)
SF‐36 Health change	0.197	0.420	0.065	T0 = T1 (BF_10_ = 0.253)
				T0 = T3 (BF_10_ = 0.232)
				T1 = T3 (BF_10_ = 0.239)

Compliance was generally good, as indicated by the number of sessions attended: participants completed an average of 13.65 sessions (SD = 5.46) for the cognitive training and 12.95 sessions (SD = 2.84) for the motor training. Results relative to the User Experience Questionnaire are summarized in Table 5. Values between −0.8 and 0.8 represent a more or less neutral evaluation of the corresponding scale, values >0.8 represent a positive evaluation and values < −0.8 represent a negative evaluation (Laugwitz et al., 2008). For further details on the User Experience Questionnaire refer to Table 5.

**TABLE 5:** Descriptive values of the dimensions assessed by the User Experience Questionnaire (Laugwitz et al., 2008), completed by participants in the present study with reference to the training software (i.e., Rehability).

Scale	Mean	SD	*N*	95% confidence interval	
Attractiveness	1.754	1.219	21	1.233	2.275
Perspicuity	1.667	1.176	21	1.164	2.170
Efficiency	1.179	0.814	21	0.830	1.527
Dependability	1.369	1.145	21	0.880	1.859
Stimulation	1.440	1.242	21	0.909	1.972
Novelty	1.333	1.441	21	0.717	1.950

## DISCUSSION

This study investigated whether motor training and combined cognitive‐motor training could improve cognitive performance and well‐being in healthy older adults. Using a robust within‐subject, double‐baseline design, we found that both training types produced improvements in several cognitive domains, including memory (RAVLT), processing speed (SDMT) and inhibition (Stroop). These gains persisted when compared to the control condition (i.e. the repeated baseline with no specific training in between), suggesting the improvements were due to the interventions and not test–retest learning. However, no significant changes were observed in mood or overall well‐being, that is none of the wellbeing subtests showed change compared to the baselines.

Aging is a global and increasingly pressing issue, with the proportion of older adults rising steadily each year (Tragaki, 2024). This demographic shift brings growing health challenges, notably dementia, which places a substantial burden on caregivers and society (Gavelin et al., 2020). One promising strategy involves emphasizing primary and secondary prevention. In this context, cognitive and motor reserves—both of which can be enhanced through mental and PA—play a key protective role and remain modifiable even in later life (Stern, 2009; Pucci et al., 2024; Wu et al., 2016).

Our findings support previous research indicating that both physical and cognitive activities can enhance cognitive function in aging populations (Basak et al., 2020; Chiu et al., 2017; Northey et al., 2018; Tetlow & Edwards, 2017). Interestingly, we found no significant differences between motor and cognitive‐motor training. This may suggest that structured engagement, rather than the specific content of the activity, drives cognitive improvements. This aligns with literature showing that PA often engages cognitive processes such as attention, planning and inhibition, even when not explicitly designed to do so (Marmeleira, 2013). Alternatively, the similar effects across training types might be due to shared elements in the delivery—such as novelty, structure and participant support—which could create non‐specific benefits. Another possibility to consider is the role of placebo‐like effects in explaining the observed cognitive improvements. Participants may have benefited simply from the expectation of improvement, or the attention received during the study, rather than the specific content of the training. This phenomenon has been documented in the cognitive training literature, where participants assigned to interventions—even those with minimal cognitive challenge—showed improvements similar to those in active training groups when expectations were high or when social interaction was involved (Foroughi et al., 2016). Masurovsky (2020) also highlights the difficulty in distinguishing true cognitive effects from placebo responses in training studies with older adults, especially in the absence of carefully matched active control groups (Masurovsky, 2020). On the other hand, some studies suggest that training‐specific improvements—particularly in tasks closely related to the training domain—can exceed placebo effects when study designs include more rigorous controls (Tsai et al., 2018). In our study, the inclusion of a double‐baseline design, serving as a control condition, helped mitigate—but did not eliminate—this possibility.

Previous research has consistently linked motor activity to cognitive enhancement in older adults, though the underlying mechanisms remain a topic of debate. Proposed explanations include both neurophysiological changes—such as increased cerebral blood flow and neurogenesis—and the cognitive demands embedded in motor tasks, which engage processes like attention, planning and coordination (Kabachkova et al., 2022; Marmeleira, 2013). Our findings raise the possibility that motor training alone may be just as effective as cognitive‐motor training in supporting cognitive function. However, given the small sample size, the study may have been underpowered to detect subtle differences between the two modalities. Future research involving larger and more diverse samples is essential to robustly compare the effectiveness of different training types. Addressing this question could clarify whether simply staying active is sufficient for cognitive maintenance, or whether the specific nature of the activity plays a critical role.

Several studies have emphasized the potential of combined cognitive and motor training to further enhance cognitive outcomes (Sipilä et al., 2021; Ten Brinke et al., 2020). However, the extent to which these cognitive gains translate into everyday benefits, such as improved mood or well‐being, remains underexplored. Despite cognitive improvements, we did not observe changes in self‐reported well‐being or mood. Several explanations are plausible. First, the intervention period may have been too short for meaningful changes in psychological outcomes to emerge. Second, the timing of the final assessment—immediately after the intervention—may not have captured delayed benefits. Third, it is possible that the instruments used (e.g., PANAS, SF‐36) were not sensitive to subtle shifts in well‐being over a brief time window. Also, cognitive performance gains and subjective well‐being do not always correlate, especially in short‐term interventions (Gale et al., 2012).

A key strength of this study is the use of a double‐baseline design serving as a control condition, which helped isolate training effects from natural variability and test–retest improvements. Also, we established a priori the risks of incorrect conclusions associated with our sample size. The BFDA showed a low probability of misleading evidence (1.5%), a rate lower than the canonical 5% of the classical *p*‐value, supporting the robustness of our findings. Additionally, delivering the intervention through home‐based serious games using such a technology that offered an accessible and engaging format with high ecological validity was probably a plus. Compliance was very good: participants were instructed to complete at least three sessions per week for 1 month, and the observed attendance rates reflected this expectation. Following the initial installation of the system in participants' homes, no further in‐person support was provided, meaning that adherence to the training schedule relied entirely on the participants' initiative. While the software was designed to be user‐friendly, some participants—especially those less familiar with technology—initially expressed hesitation. Nevertheless, user experience ratings were consistently positive across all dimensions, indicating that this type of training is well‐suited for older adults.

Although the study's results are relevant, several limitations should be acknowledged. First, the relatively small sample size may have limited the ability to detect subtle or differential effects between training types and restricts the generalizability of the findings. Future studies with larger and more diverse samples are therefore needed to clarify whether motor and cognitive‐motor training differentially affect cognitive or psychological outcomes.

Second, the interventions—particularly the motor training—were not cognitively ‘pure’, as they necessarily involved multiple cognitive and sensorimotor components, although to a lesser extent than the cognitive‐motor training. While this limits causal attribution to specific training elements, it enhances ecological validity. Future research could systematically vary cognitive and motor demands to better isolate their respective contributions.

A further limitation concerns the inclusion criteria related to the technological requirements of the intervention. Specifically, participants were required to have access to a home Wi‐Fi connection and an HDMI‐equipped television in order to use the game‐based training platform. These requirements may have biassed the sample toward individuals with higher socioeconomic status and/or greater technological familiarity, potentially limiting the representativeness of the sample. As a consequence, the generalizability of the present findings to older adults with lower access to digital resources or reduced technological literacy may be constrained. Future studies should consider alternative delivery formats or provide technological support and equipment to reduce these barriers and improve external validity.

Although our analyses did not provide evidence for differential carryover effects, the absence of a washout period prevents their complete exclusion. Future studies should therefore include an explicit washout phase to more conclusively address this issue.

Finally, future studies should include longer follow‐up periods and active control conditions to strengthen causal inference and assess the durability of training effects. Overall, the present findings suggest that accessible and engaging activities, even when not cognitively complex, may support cognitive health in aging populations.

## CONCLUSION

This study examined whether motor training and combined cognitive‐motor training delivered through serious games could enhance cognitive performance and well‐being in healthy older adults. Using a preregistered, within‐subject design with a double baseline, we found that both training approaches were associated with improvements in several cognitive domains, including memory, processing speed and inhibitory control. These gains were observed relative to repeated baseline assessments, suggesting that they were not attributable to test–retest effects. In contrast, no significant changes emerged in mood or self‐reported well‐being.

Importantly, no differential effects were found between motor and cognitive‐motor training, indicating that structured and engaging activity itself, rather than the specific cognitive complexity of the intervention, may play a central role in supporting cognitive functioning in later life. Although the small sample size and the multimodal nature of the interventions limit causal specificity, the findings highlight the potential of accessible, home‐based training programmes with high ecological validity.

In conclusion, our study showed that both motor and cognitive‐motor training led to meaningful cognitive improvements in older adults, with no evidence that one approach was superior. Such changes are relevant from a preventive perspective, as maintaining or enhancing cognitive performance in healthy aging may contribute to reducing the risk or delaying the onset of pathological cognitive decline. Although well‐being and mood did not improve in the short term, these findings underscore the cognitive benefits of staying physically and mentally active. Interventions that blend physical engagement with cognitive stimulation may offer a practical and enjoyable strategy to promote healthy aging.

## AUTHOR CONTRIBUTIONS

**Silvia Gobbo:** Conceptualization; data curation; formal analysis; investigation; methodology; writing – original draft; writing – review and editing. **Elisa Galli:** Formal analysis; investigation; visualization; writing – review and editing. **Daniele Romano:** Conceptualization; data curation; formal analysis; funding acquisition; methodology; project administration; supervision; visualization; writing – review and editing.

## FUNDING INFORMATION

The present study was funded within the MUSA—Multilayered Urban Sustainability Action—project, funded by the European Union—NextGenerationEU, under the National Recovery and Resilience Plan (NRRP) Mission 4 Component 2 Investment Line 1.5: Strengthening of research structures and creation of R&D ‘innovation ecosystems’, set up of ‘territorial leaders in R&D’.

## CONFLICT OF INTEREST STATEMENT

The authors report there are no competing interests to declare.

## Supporting information


Data S1.

## Data Availability

Hypotheses and data analysis plan were pre‐registered in aspredicted.com (https://aspredicted.org/s73s‐bz8k.pdf). Data and statistical analyses are made open and can be found in OSF: https://osf.io/8rmak/?view_only=63b58cc83b1f43ff8507c28845b8f095.
