# Supplementary material for: Effects of motor and cognitive‐motor training on cognitive performance in healthy older adults
Source: J Neuropsychol. 2026 Mar 20;20(3):517–32. doi: 10.1111/jnp.70043 (PMC13552247; doi:10.1111/jnp.70043)
Supplement: Supplementary file 1 [file JNP-20-517-s001.docx]

**Summary**

1. **Pre-registered analyses results**
   1.1. **Cognitive tests**
        1.1.1. **Moca**
        1.1.2. **RAVLT immediate recall**
        1.1.3. **RAVLT delayed recall**
        1.1.4. **Stroop test (time)**
        1.1.5. **Symbol Digit Modalities Test (SDMT)**
        1.1.6. **Trail Making Test A (TMT-A)**
        1.1.7. **Trail Making Test B (TMT-B)**
        1.1.8. **Trail Making Test B-A (TMT-BA)**
   1.2. **Well-being**
        1.2.1. **Positive And Negative Affect Scale – Positive (PANAS positive)**
        1.2.2. **Positive And Negative Affect Scale – Negative (PANAS negative)**
        1.2.3. **SF-36 Physical functioning**
        1.2.4. **SF-36 Role of limitation due to physical health**
        1.2.5. **SF-36 Role of limitations due to emotional problems**
        1.2.6. **SF-36 Energy/Fatigue**
        1.2.7. **SF-36 Emotional well-being**
        1.2.8. **SF-36 Social functioning**
        1.2.9. **SF-36 Pain**
        1.2.10. **SF-36 General Health**
        1.2.11. **SF-36 Health change**
2. **Complete result tables of the analyses of the main text**
   2.1. **Cognitive tests**
        2.1.1. **Moca**
        2.1.2. **RAVLT immediate recall**
        2.1.3. **RAVLT delayed recall**
        2.1.4. **Stroop test (time)**
        2.1.5. **Symbol Digit Modalities Test (SDMT)**
        2.1.6. **Trail Making Test A (TMT-A)**
        2.1.7. **Trail Making Test B (TMT-B)**
        2.1.8. **Trail Making Test B-A (TMT-BA)**
   2.2. **Well-being**
        2.2.1. **Positive And Negative Affect Scale – Positive (PANAS positive)**
        2.2.2. **Positive And Negative Affect Scale – Negative (PANAS negative)**
        2.2.3. **SF-36 Physical functioning**
        2.2.4. **SF-36 Role of limitation due to physical health**
        2.2.5. **SF-36 Role of limitations due to emotional problems**
        2.2.6. **SF-36 Energy/Fatigue**
        2.2.7. **SF-36 Emotional well-being**
        2.2.8. **SF-36 Social functioning**
        2.2.9. **SF-36 Pain**
        2.2.10. **SF-36 General Health**
        2.2.11. **SF-36 Health change**

Results relative to the pre-registered and explorative analyses are reported below. For each test we report the model comparison table, the analysis of effect table and post-hoc comparisons.

1. **Pre-registered analyses**
   1.1. **Cognitive tests**
        1.1.1. ***Moca***

| *Model Comparison* | | | | | | | | | | | | | | | | | | |
| --- | --- | --- | --- | --- | --- | --- | --- | --- | --- | --- | --- | --- | --- | --- | --- | --- | --- | --- |
| Models | | | | | | | | | P(M) | | P(M\|data) | | BF_M_ | | BF_10_ | | error % | |
| Null model (incl. CRIqtot, MRIq, subject, and random slopes) | | | | | | | |  | 0.500 |  | 0.827 |  | 4.794 |  | 1.000 |  |  |  |
| Training | | | | | | | |  | 0.500 |  | 0.173 |  | 0.209 |  | 0.209 |  | 3.637 |  |
|  | | | | | | | | | | | | | | | | | | |
|  | | | | | | | | | | | | | | | | | | |
| *Analysis of Effects* | | | | | | | | | | | | | | | | | | |
| Effects | | P(incl) | | P(excl) | | P(incl\|data) | | P(excl\|data) | | BF_incl_ | |  |  |  |  |  |  |  |
| Training |  | 0.500 |  | 0.500 |  | 0.173 |  | 0.827 |  | 0.209 |  |  |  |  |  |  |  |  |
|  | | | | | | | | | | | |  |  |  |  |  |  |  |

| *Post Hoc Comparisons - Training* | | | | | | | | | | | |  |  |  |  |  |
| --- | --- | --- | --- | --- | --- | --- | --- | --- | --- | --- | --- | --- | --- | --- | --- | --- |
|  | |  | | Prior Odds | | Posterior Odds | | BF_10, U_ | | error % | |  |  |  |  |  |
| Baseline |  | Motor |  | 0.587 |  | 0.294 |  | 0.500 |  | 0.023 |  |  |  |  |  |  |
|  |  | Cognitive |  | 0.587 |  | 0.139 |  | 0.236 |  | 0.018 |  |  |  |  |  |  |
| Motor |  | Cognitive |  | 0.587 |  | 0.164 |  | 0.279 |  | 0.019 |  |  |  |  |  |  |
|  | | | | | | | | | | | |  |  |  |  |  |
| 1.1.2. **RAVLT immediate recall** | | | | | | | | | | | |  |  |  |  |  |
| *Model Comparison* | | | | | | | | | | | |  |  |  |  |  |
| Models | | | | | | | P(M) | | P(M\|data) | | BF_M_ | | BF_10_ | | error % | |
| Training | | | | | |  | 0.500 |  | 0.996 |  | 244.453 |  | 1.000 |  |  |  |
| Null model (incl. CRIqtot, MRIq, subject, and random slopes) | | | | | |  | 0.500 |  | 0.004 |  | 0.004 |  | 0.004 |  | 2.081 |  |
|  | | | | | | | | | | | | | | | | |

| *Analysis of Effects* | | | | | | | | | | | |
| --- | --- | --- | --- | --- | --- | --- | --- | --- | --- | --- | --- |
| Effects | | P(incl) | | P(excl) | | P(incl\|data) | | P(excl\|data) | | BF_incl_ | |
| Training |  | 0.500 |  | 0.500 |  | 0.996 |  | 0.004 |  | 244.453 |  |
|  | | | | | | | | | | | |

| *Post Hoc Comparisons - Training* | | | | | | | | | | | |
| --- | --- | --- | --- | --- | --- | --- | --- | --- | --- | --- | --- |
|  | |  | | Prior Odds | | Posterior Odds | | BF_10, U_ | | error % | |
| Baseline |  | Cognitive |  | 0.587 |  | 8.304 |  | 14.137 |  | 2.435×10^-7^ |  |
|  |  | Motor |  | 0.587 |  | 9.071 |  | 15.442 |  | 2.544×10^-7^ |  |
| Cognitive |  | Motor |  | 0.587 |  | 0.136 |  | 0.232 |  | 0.017 |  |
|  | | | | | | | | | | | |
|  | | | | | | | | | | | |

1.1.3. **RAVLT delayed recall**

| *Model Comparison* | | | | | | | | | | | |
| --- | --- | --- | --- | --- | --- | --- | --- | --- | --- | --- | --- |
| Models | | P(M) | | P(M\|data) | | BF_M_ | | BF_10_ | | error % | |
| Training |  | 0.500 |  | 0.987 |  | 74.813 |  | 1.000 |  |  |  |
| Null model (incl. CRIqtot, MRIq, subject, and random slopes) |  | 0.500 |  | 0.013 |  | 0.013 |  | 0.013 |  | 1.288 |  |
|  | | | | | | | | | | | |
| *Analysis of Effects* \| \| \| \| \| \| \| \| \| \| \|; Effects \| \| P(incl) \| \| P(excl) \| \| P(incl\|data) \| \| P(excl\|data) \| \| BF_incl_ \|; Training \| \| 0.500 \| \| 0.500 \| \| 0.987 \| \| 0.013 \| \| 74.813 \| *Post Hoc Comparisons - Training* \| \| \| \| \| \| \| \| \| \| \|; \| \| \| \| Prior Odds \| \| Posterior Odds \| \| BF_10, U_ \| \| error % \|; Baseline \| \| Cognitive \| \| 0.587 \| \| 8.241 \| \| 14.030 \| \| 2.426×10^-7^ \|; \| \| Motor \| \| 0.587 \| \| 3.483 \| \| 5.929 \| \| 2.558×10^-7^ \|; Cognitive \| \| Motor \| \| 0.587 \| \| 0.161 \| \| 0.274 \| \| 0.019 \| | | | | | | | | | | | |

1.1.4. **Stroop test (time)**

| *Model Comparison* | | | | | | | | | | | |
| --- | --- | --- | --- | --- | --- | --- | --- | --- | --- | --- | --- |
| Models | | P(M) | | P(M\|data) | | BF_M_ | | BF_10_ | | error % | |
| Null model (incl. CRIqtot, MRIq, subject, and random slopes) |  | 0.500 |  | 0.802 |  | 4.047 |  | 1.000 |  |  |  |
| Training |  | 0.500 |  | 0.198 |  | 0.247 |  | 0.247 |  | 1.968 |  |
|  | | | | | | | | | | | |
| *Analysis of Effects* \| \| \| \| \| \| \| \| \| \| \|; Effects \| \| P(incl) \| \| P(excl) \| \| P(incl\|data) \| \| P(excl\|data) \| \| BF_incl_ \|; Training \| \| 0.500 \| \| 0.500 \| \| 0.198 \| \| 0.802 \| \| 0.247 \| *Post Hoc Comparisons - Training* \| \| \| \| \| \| \| \| \| \| \|; \| \| \| \| Prior Odds \| \| Posterior Odds \| \| BF_10, U_ \| \| error % \|; Baseline \| \| Cognitive \| \| 0.587 \| \| 0.188 \| \| 0.320 \| \| 0.019 \|; \| \| Motor \| \| 0.587 \| \| 0.140 \| \| 0.238 \| \| 0.016 \|; Cognitive \| \| Motor \| \| 0.587 \| \| 0.263 \| \| 0.447 \| \| 0.021 \| | | | | | | | | | | | |

1.1.5. **SDMT**

| *Model Comparison* | | | | | | | | | | | |
| --- | --- | --- | --- | --- | --- | --- | --- | --- | --- | --- | --- |
| Models | | P(M) | | P(M\|data) | | BF_M_ | | BF_10_ | | error % | |
| Null model (incl. CRIqtot, MRIq, subject, and random slopes) |  | 0.500 |  | 0.808 |  | 4.212 |  | 1.000 |  |  |  |
| Training |  | 0.500 |  | 0.192 |  | 0.237 |  | 0.237 |  | 1.934 |  |
|  | | | | | | | | | | | |
| *Analysis of Effects* \| \| \| \| \| \| \| \| \| \| \|; Effects \| \| P(incl) \| \| P(excl) \| \| P(incl\|data) \| \| P(excl\|data) \| \| BF_incl_ \|; Training \| \| 0.500 \| \| 0.500 \| \| 0.192 \| \| 0.808 \| \| 0.237 \| | | | | | | | | | | | |

| *Post Hoc Comparisons - Training* | | | | | | | | | | | |
| --- | --- | --- | --- | --- | --- | --- | --- | --- | --- | --- | --- |
|  | |  | | Prior Odds | | Posterior Odds | | BF_10, U_ | | error % | |
| Baseline |  | Cognitive |  | 0.587 |  | 0.252 |  | 0.429 |  | 0.022 |  |
|  |  | Motor |  | 0.587 |  | 0.140 |  | 0.238 |  | 0.018 |  |
| Cognitive |  | Motor |  | 0.587 |  | 0.174 |  | 0.297 |  | 0.020 |  |
|  | | | | | | | | | | | |
|  | | | | | | | | | | | |

1.1.6. **TMT-A**

| *Model Comparison* | | | | | | | | | | | |
| --- | --- | --- | --- | --- | --- | --- | --- | --- | --- | --- | --- |
| Models | | P(M) | | P(M\|data) | | BF_M_ | | BF_10_ | | error % | |
| Null model (incl. CRIqtot, MRIq, subject, and random slopes) |  | 0.500 |  | 0.756 |  | 3.096 |  | 1.000 |  |  |  |
| Training |  | 0.500 |  | 0.244 |  | 0.323 |  | 0.323 |  | 2.574 |  |
|  | | | | | | | | | | | |
| *Analysis of Effects* \| \| \| \| \| \| \| \| \| \| \|; Effects \| \| P(incl) \| \| P(excl) \| \| P(incl\|data) \| \| P(excl\|data) \| \| BF_incl_ \|; Training \| \| 0.500 \| \| 0.500 \| \| 0.244 \| \| 0.756 \| \| 0.323 \| *Post Hoc Comparisons - Training* \| \| \| \| \| \| \| \| \| \| \|; \| \| \| \| Prior Odds \| \| Posterior Odds \| \| BF_10, U_ \| \| error % \|; Baseline \| \| Cognitive \| \| 0.587 \| \| 0.164 \| \| 0.279 \| \| 0.019 \|; \| \| Motor \| \| 0.587 \| \| 0.349 \| \| 0.594 \| \| 0.023 \|; Cognitive \| \| Motor \| \| 0.587 \| \| 0.167 \| \| 0.284 \| \| 0.019 \| | | | | | | | | | | | |

1.1.7. **TMT-B**

| *Model Comparison* | | | | | | | | | | | |
| --- | --- | --- | --- | --- | --- | --- | --- | --- | --- | --- | --- |
| Models | | P(M) | | P(M\|data) | | BF_M_ | | BF_10_ | | error % | |
| Null model (incl. CRIqtot, MRIq, subject, and random slopes) |  | 0.500 |  | 0.549 |  | 1.218 |  | 1.000 |  |  |  |
| Training |  | 0.500 |  | 0.451 |  | 0.821 |  | 0.821 |  | 2.461 |  |
|  | | | | | | | | | | | |
| *Analysis of Effects* \| \| \| \| \| \| \| \| \| \| \|; Effects \| \| P(incl) \| \| P(excl) \| \| P(incl\|data) \| \| P(excl\|data) \| \| BF_incl_ \|; Training \| \| 0.500 \| \| 0.500 \| \| 0.451 \| \| 0.549 \| \| 0.821 \| *Post Hoc Comparisons - Training* \| \| \| \| \| \| \| \| \| \| \|; \| \| \| \| Prior Odds \| \| Posterior Odds \| \| BF_10, U_ \| \| error % \|; Baseline \| \| Cognitive \| \| 0.587 \| \| 0.400 \| \| 0.681 \| \| 0.024 \|; \| \| Motor \| \| 0.587 \| \| 0.209 \| \| 0.356 \| \| 0.021 \|; Cognitive \| \| Motor \| \| 0.587 \| \| 0.228 \| \| 0.389 \| \| 0.021 \| | | | | | | | | | | | |

1.1.8. **TMT-BA**

| *Model Comparison* | | | | | | | | | | | |
| --- | --- | --- | --- | --- | --- | --- | --- | --- | --- | --- | --- |
| Models | | P(M) | | P(M\|data) | | BF_M_ | | BF_10_ | | error % | |
| Null model (incl. CRIqtot, MRIq, subject, and random slopes) |  | 0.500 |  | 0.567 |  | 1.310 |  | 1.000 |  |  |  |
| Training |  | 0.500 |  | 0.433 |  | 0.763 |  | 0.763 |  | 1.253 |  |
|  | | | | | | | | | | | |
| *Analysis of Effects* \| \| \| \| \| \| \| \| \| \| \|; Effects \| \| P(incl) \| \| P(excl) \| \| P(incl\|data) \| \| P(excl\|data) \| \| BF_incl_ \|; Training \| \| 0.500 \| \| 0.500 \| \| 0.433 \| \| 0.567 \| \| 0.763 \| *Post Hoc Comparisons - Training* \| \| \| \| \| \| \| \| \| \| \|; \| \| \| \| Prior Odds \| \| Posterior Odds \| \| BF_10, U_ \| \| error % \|; Baseline \| \| Cognitive \| \| 0.587 \| \| 0.374 \| \| 0.637 \| \| 0.023 \|; \| \| Motor \| \| 0.587 \| \| 0.165 \| \| 0.281 \| \| 0.019 \|; Cognitive \| \| Motor \| \| 0.587 \| \| 0.295 \| \| 0.501 \| \| 0.023 \| | | | | | | | | | | | |

1.2. **Well-being**
     1.2.1. **PANAS positive**

| *Model Comparison* | | | | | | | | | | | |
| --- | --- | --- | --- | --- | --- | --- | --- | --- | --- | --- | --- |
| Models | | P(M) | | P(M\|data) | | BF_M_ | | BF_10_ | | error % | |
| Null model (incl. CRIqtot, MRIq, subject, and random slopes) |  | 0.500 |  | 0.815 |  | 4.402 |  | 1.000 |  |  |  |
| Training |  | 0.500 |  | 0.185 |  | 0.227 |  | 0.227 |  | 2.543 |  |
|  | | | | | | | | | | | |
| *Analysis of Effects* \| \| \| \| \| \| \| \| \| \| \|; Effects \| \| P(incl) \| \| P(excl) \| \| P(incl\|data) \| \| P(excl\|data) \| \| BF_incl_ \|; Training \| \| 0.500 \| \| 0.500 \| \| 0.185 \| \| 0.815 \| \| 0.227 \| *Post Hoc Comparisons - Training* \| \| \| \| \| \| \| \| \| \| \|; \| \| \| \| Prior Odds \| \| Posterior Odds \| \| BF_10, U_ \| \| error % \|; Baseline \| \| Cognitive \| \| 0.587 \| \| 0.201 \| \| 0.342 \| \| 0.021 \|; \| \| Motor \| \| 0.587 \| \| 0.173 \| \| 0.295 \| \| 0.020 \|; Cognitive \| \| Motor \| \| 0.587 \| \| 0.147 \| \| 0.250 \| \| 0.018 \| | | | | | | | | | | | |

1.2.2. **PANAS negative**

| *Model Comparison* | | | | | | | | | | | |
| --- | --- | --- | --- | --- | --- | --- | --- | --- | --- | --- | --- |
| Models | | P(M) | | P(M\|data) | | BF_M_ | | BF_10_ | | error % | |
| Null model (incl. CRIqtot, MRIq, subject, and random slopes) |  | 0.500 |  | 0.845 |  | 5.455 |  | 1.000 |  |  |  |
| Training |  | 0.500 |  | 0.155 |  | 0.183 |  | 0.183 |  | 2.307 |  |
|  | | | | | | | | | | | |
| *Analysis of Effects* \| \| \| \| \| \| \| \| \| \| \|; Effects \| \| P(incl) \| \| P(excl) \| \| P(incl\|data) \| \| P(excl\|data) \| \| BF_incl_ \|; Training \| \| 0.500 \| \| 0.500 \| \| 0.155 \| \| 0.845 \| \| 0.183 \| *Post Hoc Comparisons - Training* \| \| \| \| \| \| \| \| \| \| \|; \| \| \| \| Prior Odds \| \| Posterior Odds \| \| BF_10, U_ \| \| error % \|; Baseline \| \| Cognitive \| \| 0.587 \| \| 0.149 \| \| 0.254 \| \| 0.018 \|; \| \| Motor \| \| 0.587 \| \| 0.149 \| \| 0.253 \| \| 0.018 \|; Cognitive \| \| Motor \| \| 0.587 \| \| 0.169 \| \| 0.288 \| \| 0.019 \| | | | | | | | | | | | |

1.2.3. **SF-36 Physical functioning**

| *Model Comparison* | | | | | | | | | | | |
| --- | --- | --- | --- | --- | --- | --- | --- | --- | --- | --- | --- |
| Models | | P(M) | | P(M\|data) | | BF_M_ | | BF_10_ | | error % | |
| Null model (incl. CRIqtot, MRIq, subject, and random slopes) |  | 0.500 |  | 0.845 |  | 5.456 |  | 1.000 |  |  |  |
| Training |  | 0.500 |  | 0.155 |  | 0.183 |  | 0.183 |  | 7.317 |  |
|  | | | | | | | | | | | |
| *Analysis of Effects* \| \| \| \| \| \| \| \| \| \| \|; Effects \| \| P(incl) \| \| P(excl) \| \| P(incl\|data) \| \| P(excl\|data) \| \| BF_incl_ \|; Training \| \| 0.500 \| \| 0.500 \| \| 0.155 \| \| 0.845 \| \| 0.183 \| | | | | | | | | | | | |

| *Post Hoc Comparisons - Training* | | | | | | | | | | | |
| --- | --- | --- | --- | --- | --- | --- | --- | --- | --- | --- | --- |
|  | |  | | Prior Odds | | Posterior Odds | | BF_10, U_ | | error % | |
| Baseline |  | Cognitive |  | 0.587 |  | 0.139 |  | 0.236 |  | 0.018 |  |
|  |  | Motor |  | 0.587 |  | 0.160 |  | 0.272 |  | 0.019 |  |
| Cognitive |  | Motor |  | 0.587 |  | 0.146 |  | 0.249 |  | 0.018 |  |
|  | | | | | | | | | | | |

1.2.4. **SF-36 Role of limitation due to physical health**

| *Model Comparison* | | | | | | | | | | | |
| --- | --- | --- | --- | --- | --- | --- | --- | --- | --- | --- | --- |
| Models | | P(M) | | P(M\|data) | | BF_M_ | | BF_10_ | | error % | |
| Null model (incl. CRIqtot, MRIq, subject, and random slopes) |  | 0.500 |  | 0.855 |  | 5.886 |  | 1.000 |  |  |  |
| Training |  | 0.500 |  | 0.145 |  | 0.170 |  | 0.170 |  | 1.399 |  |
|  | | | | | | | | | | | |

| *Analysis of Effects* | | | | | | | | | | | |
| --- | --- | --- | --- | --- | --- | --- | --- | --- | --- | --- | --- |
| Effects | | P(incl) | | P(excl) | | P(incl\|data) | | P(excl\|data) | | BF_incl_ | |
| Training |  | 0.500 |  | 0.500 |  | 0.145 |  | 0.855 |  | 0.170 |  |
|  | | | | | | | | | | | |

| *Post Hoc Comparisons - Training* | | | | | | | | | | | |
| --- | --- | --- | --- | --- | --- | --- | --- | --- | --- | --- | --- |
|  | |  | | Prior Odds | | Posterior Odds | | BF_10, U_ | | error % | |
| Baseline |  | Cognitive |  | 0.587 |  | 0.146 |  | 0.249 |  | 0.018 |  |
|  |  | Motor |  | 0.587 |  | 0.174 |  | 0.297 |  | 0.020 |  |
| Cognitive |  | Motor |  | 0.587 |  | 0.140 |  | 0.238 |  | 0.018 |  |
|  | | | | | | | | | | | |
|  | | | | | | | | | | | |

1.2.5. **SF-36 Role of limitations due to emotional problems**

| *Model Comparison* | | | | | | | | | | | |
| --- | --- | --- | --- | --- | --- | --- | --- | --- | --- | --- | --- |
| Models | | P(M) | | P(M\|data) | | BF_M_ | | BF_10_ | | error % | |
| Null model (incl. CRIqtot, MRIq, subject, and random slopes) |  | 0.500 |  | 0.818 |  | 4.501 |  | 1.000 |  |  |  |
| Training |  | 0.500 |  | 0.182 |  | 0.222 |  | 0.222 |  | 5.287 |  |
|  | | | | | | | | | | | |

| *Analysis of Effects* | | | | | | | | | | | |
| --- | --- | --- | --- | --- | --- | --- | --- | --- | --- | --- | --- |
| Effects | | P(incl) | | P(excl) | | P(incl\|data) | | P(excl\|data) | | BF_incl_ | |
| Training |  | 0.500 |  | 0.500 |  | 0.182 |  | 0.818 |  | 0.222 |  |
|  | | | | | | | | | | | |

| *Post Hoc Comparisons - Training* | | | | | | | | | | | |
| --- | --- | --- | --- | --- | --- | --- | --- | --- | --- | --- | --- |
|  | |  | | Prior Odds | | Posterior Odds | | BF_10, U_ | | error % | |
| Baseline |  | Cognitive |  | 0.587 |  | 0.136 |  | 0.232 |  | 0.017 |  |
|  |  | Motor |  | 0.587 |  | 0.188 |  | 0.320 |  | 0.020 |  |
| Cognitive |  | Motor |  | 0.587 |  | 0.202 |  | 0.344 |  | 0.021 |  |
|  | | | | | | | | | | | |
|  | | | | | | | | | | | |

1.2.6. **SF-36 Energy/Fatigue**

| *Model Comparison* | | | | | | | | | | | |
| --- | --- | --- | --- | --- | --- | --- | --- | --- | --- | --- | --- |
| Models | | P(M) | | P(M\|data) | | BF_M_ | | BF_10_ | | error % | |
| Null model (incl. CRIqtot, MRIq, subject, and random slopes) |  | 0.500 |  | 0.789 |  | 3.743 |  | 1.000 |  |  |  |
| Training |  | 0.500 |  | 0.211 |  | 0.267 |  | 0.267 |  | 2.187 |  |
|  | | | | | | | | | | | |

| *Analysis of Effects* | | | | | | | | | | | |
| --- | --- | --- | --- | --- | --- | --- | --- | --- | --- | --- | --- |
| Effects | | P(incl) | | P(excl) | | P(incl\|data) | | P(excl\|data) | | BF_incl_ | |
| Training |  | 0.500 |  | 0.500 |  | 0.211 |  | 0.789 |  | 0.267 |  |
|  | | | | | | | | | | | |

| *Post Hoc Comparisons - Training* | | | | | | | | | | | |
| --- | --- | --- | --- | --- | --- | --- | --- | --- | --- | --- | --- |
|  | |  | | Prior Odds | | Posterior Odds | | BF_10, U_ | | error % | |
| Baseline |  | Cognitive |  | 0.587 |  | 0.137 |  | 0.234 |  | 0.018 |  |
|  |  | Motor |  | 0.587 |  | 0.243 |  | 0.414 |  | 0.022 |  |
| Cognitive |  | Motor |  | 0.587 |  | 0.211 |  | 0.360 |  | 0.021 |  |
|  | | | | | | | | | | | |
|  | | | | | | | | | | | |

1.2.7. **SF-36 Emotional well-being**

| *Model Comparison* | | | | | | | | | | | |
| --- | --- | --- | --- | --- | --- | --- | --- | --- | --- | --- | --- |
| Models | | P(M) | | P(M\|data) | | BF_M_ | | BF_10_ | | error % | |
| Null model (incl. CRIqtot, MRIq, subject, and random slopes) |  | 0.500 |  | 0.764 |  | 3.234 |  | 1.000 |  |  |  |
| Training |  | 0.500 |  | 0.236 |  | 0.309 |  | 0.309 |  | 1.613 |  |
|  | | | | | | | | | | | |
| *Analysis of Effects* \| \| \| \| \| \| \| \| \| \| \|; Effects \| \| P(incl) \| \| P(excl) \| \| P(incl\|data) \| \| P(excl\|data) \| \| BF_incl_ \|; Training \| \| 0.500 \| \| 0.500 \| \| 0.236 \| \| 0.764 \| \| 0.309 \| *Post Hoc Comparisons - Training* \| \| \| \| \| \| \| \| \| \| \|; \| \| \| \| Prior Odds \| \| Posterior Odds \| \| BF_10, U_ \| \| error % \|; Baseline \| \| Cognitive \| \| 0.587 \| \| 0.140 \| \| 0.239 \| \| 0.018 \|; \| \| Motor \| \| 0.587 \| \| 0.204 \| \| 0.347 \| \| 0.021 \|; Cognitive \| \| Motor \| \| 0.587 \| \| 0.295 \| \| 0.501 \| \| 0.023 \| | | | | | | | | | | | |

1.2.8. **SF-36 Social functioning**

| *Model Comparison* | | | | | | | | | | | |
| --- | --- | --- | --- | --- | --- | --- | --- | --- | --- | --- | --- |
| Models | | P(M) | | P(M\|data) | | BF_M_ | | BF_10_ | | error % | |
| Null model (incl. CRIqtot, MRIq, subject, and random slopes) |  | 0.500 |  | 0.772 |  | 3.392 |  | 1.000 |  |  |  |
| Training |  | 0.500 |  | 0.228 |  | 0.295 |  | 0.295 |  | 1.494 |  |
|  | | | | | | | | | | | |

| *Analysis of Effects* | | | | | | | | | | | |
| --- | --- | --- | --- | --- | --- | --- | --- | --- | --- | --- | --- |
| Effects | | P(incl) | | P(excl) | | P(incl\|data) | | P(excl\|data) | | BF_incl_ | |
| Training |  | 0.500 |  | 0.500 |  | 0.228 |  | 0.772 |  | 0.295 |  |
|  | | | | | | | | | | | |

| *Post Hoc Comparisons - Training* | | | | | | | | | | | |
| --- | --- | --- | --- | --- | --- | --- | --- | --- | --- | --- | --- |
|  | |  | | Prior Odds | | Posterior Odds | | BF_10, U_ | | error % | |
| Baseline |  | Cognitive |  | 0.587 |  | 0.137 |  | 0.233 |  | 0.018 |  |
|  |  | Motor |  | 0.587 |  | 0.273 |  | 0.465 |  | 0.022 |  |
| Cognitive |  | Motor |  | 0.587 |  | 0.187 |  | 0.319 |  | 0.020 |  |
|  | | | | | | | | | | | |

1.2.9. **SF-36 Pain**

| *Model Comparison* | | | | | | | | | | | |
| --- | --- | --- | --- | --- | --- | --- | --- | --- | --- | --- | --- |
| Models | | P(M) | | P(M\|data) | | BF_M_ | | BF_10_ | | error % | |
| Null model (incl. CRIqtot, MRIq, subject, and random slopes) |  | 0.500 |  | 0.633 |  | 1.723 |  | 1.000 |  |  |  |
| Training |  | 0.500 |  | 0.367 |  | 0.581 |  | 0.581 |  | 1.835 |  |
|  | | | | | | | | | | | |

| *Analysis of Effects* | | | | | | | | | | | |
| --- | --- | --- | --- | --- | --- | --- | --- | --- | --- | --- | --- |
| Effects | | P(incl) | | P(excl) | | P(incl\|data) | | P(excl\|data) | | BF_incl_ | |
| Training |  | 0.500 |  | 0.500 |  | 0.367 |  | 0.633 |  | 0.581 |  |
|  | | | | | | | | | | | |

| *Post Hoc Comparisons - Training* | | | | | | | | | | | |
| --- | --- | --- | --- | --- | --- | --- | --- | --- | --- | --- | --- |
|  | |  | | Prior Odds | | Posterior Odds | | BF_10, U_ | | error % | |
| Baseline |  | Cognitive |  | 0.587 |  | 0.270 |  | 0.459 |  | 0.022 |  |
|  |  | Motor |  | 0.587 |  | 0.542 |  | 0.923 |  | 0.024 |  |
| Cognitive |  | Motor |  | 0.587 |  | 0.282 |  | 0.481 |  | 0.023 |  |
|  | | | | | | | | | | | |
| 1.2.10. **SF-36 General Health** *Model Comparison* \| \| \| \| \| \| \| \| \| \| \|; Models \| \| P(M) \| \| P(M\|data) \| \| BF_M_ \| \| BF_10_ \| \| error % \|; Null model (incl. CRIqtot, MRIq, subject, and random slopes) \| \| 0.500 \| \| 0.559 \| \| 1.269 \| \| 1.000 \| \| \|; Training \| \| 0.500 \| \| 0.441 \| \| 0.788 \| \| 0.788 \| \| 1.263 \| *Analysis of Effects* \| \| \| \| \| \| \| \| \| \| \|; Effects \| \| P(incl) \| \| P(excl) \| \| P(incl\|data) \| \| P(excl\|data) \| \| BF_incl_ \|; Training \| \| 0.500 \| \| 0.500 \| \| 0.441 \| \| 0.559 \| \| 0.788 \| | | | | | | | | | | | |

| *Post Hoc Comparisons - Training* | | | | | | | | | | | |
| --- | --- | --- | --- | --- | --- | --- | --- | --- | --- | --- | --- |
|  | |  | | Prior Odds | | Posterior Odds | | BF_10, U_ | | error % | |
| Baseline |  | Cognitive |  | 0.587 |  | 0.666 |  | 1.134 |  | 0.024 |  |
|  |  | Motor |  | 0.587 |  | 0.296 |  | 0.504 |  | 0.023 |  |
| Cognitive |  | Motor |  | 0.587 |  | 0.149 |  | 0.254 |  | 0.018 |  |
|  | | | | | | | | | | | |
|  | | | | | | | | | | | |

1.2.11. **SF-36 Health change**

| *Model Comparison* | | | | | | | | | | | |
| --- | --- | --- | --- | --- | --- | --- | --- | --- | --- | --- | --- |
| Models | | P(M) | | P(M\|data) | | BF_M_ | | BF_10_ | | error % | |
| Null model (incl. CRIqtot, MRIq, subject, and random slopes) |  | 0.500 |  | 0.854 |  | 5.867 |  | 1.000 |  |  |  |
| Training |  | 0.500 |  | 0.146 |  | 0.170 |  | 0.170 |  | 3.346 |  |
|  | | | | | | | | | | | |

| *Analysis of Effects* | | | | | | | | | | | |
| --- | --- | --- | --- | --- | --- | --- | --- | --- | --- | --- | --- |
| Effects | | P(incl) | | P(excl) | | P(incl\|data) | | P(excl\|data) | | BF_incl_ | |
| Training |  | 0.500 |  | 0.500 |  | 0.146 |  | 0.854 |  | 0.170 |  |
|  | | | | | | | | | | | |

| *Post Hoc Comparisons - Training* | | | | | | | | | | | |
| --- | --- | --- | --- | --- | --- | --- | --- | --- | --- | --- | --- |
|  | |  | | Prior Odds | | Posterior Odds | | BF_10, U_ | | error % | |
| Baseline |  | Cognitive |  | 0.587 |  | 0.172 |  | 0.293 |  | 0.020 |  |
|  |  | Motor |  | 0.587 |  | 0.136 |  | 0.232 |  | 0.017 |  |
| Cognitive |  | Motor |  | 0.587 |  | 0.151 |  | 0.257 |  | 0.018 |  |
|  | | | | | | | | | | | |
|  | | | | | | | | | | | |

2. **Explorative analyses**
   2.1. **Cognitive tests**
        2.1.1. **Moca**

| *Model Comparison* | | | | | | | | | | | |
| --- | --- | --- | --- | --- | --- | --- | --- | --- | --- | --- | --- |
| Models | | P(M) | | P(M\|data) | | BF_M_ | | BF_10_ | | error % | |
| timepoint |  | 0.200 |  | 0.508 |  | 4.133 |  | 1.000 |  |  |  |
| timepoint + Ordine |  | 0.200 |  | 0.278 |  | 1.542 |  | 0.547 |  | 3.903 |  |
| Null model (incl. CRIq_tot, MRIq, subject, and random slopes) |  | 0.200 |  | 0.096 |  | 0.424 |  | 0.189 |  | 2.814 |  |
| timepoint + Ordine + timepoint ✻  Ordine |  | 0.200 |  | 0.066 |  | 0.282 |  | 0.129 |  | 4.502 |  |
| Ordine |  | 0.200 |  | 0.052 |  | 0.219 |  | 0.102 |  | 3.691 |  |
|  | | | | | | | | | | | |

| *Analysis of Effects* | | | | | | | | | | | |
| --- | --- | --- | --- | --- | --- | --- | --- | --- | --- | --- | --- |
| Effects | | P(incl) | | P(excl) | | P(incl\|data) | | P(excl\|data) | | BF_incl_ | |
| timepoint |  | 0.600 |  | 0.400 |  | 0.852 |  | 0.148 |  | 3.844 |  |
| Ordine |  | 0.600 |  | 0.400 |  | 0.396 |  | 0.604 |  | 0.437 |  |
| timepoint ✻  Ordine |  | 0.200 |  | 0.800 |  | 0.066 |  | 0.934 |  | 0.282 |  |
|  | | | | | | | | | | | |

| *Post Hoc Comparisons - timepoint* | | | | | | | | | | | |
| --- | --- | --- | --- | --- | --- | --- | --- | --- | --- | --- | --- |
|  | |  | | Prior Odds | | Posterior Odds | | BF_10, U_ | | error % | |
| 0 |  | 1 |  | 0.587 |  | 1.294 |  | 2.202 |  | 1.327×10^-6^ |  |
|  |  | 3 |  | 0.587 |  | 2.770 |  | 4.716 |  | 3.214×10^-7^ |  |
| 1 |  | 3 |  | 0.587 |  | 0.183 |  | 0.311 |  | 0.019 |  |
|  | | | | | | | | | | | |
|  | | | | | | | | | | | |

| *Post Hoc Comparisons - Ordine* | | | | | | | | | | | |
| --- | --- | --- | --- | --- | --- | --- | --- | --- | --- | --- | --- |
|  | |  | | Prior Odds | | Posterior Odds | | BF_10, U_ | | error % | |
| C-M |  | M-C |  | 1.000 |  | 0.269 |  | 0.269 |  | 0.009 |  |
|  | | | | | | | | | | | |
|  | | | | | | | | | | | |

2.1.2. **RAVLT immediate recall**

| *Model Comparison* | | | | | | | | | | | | | | | | |  |
| --- | --- | --- | --- | --- | --- | --- | --- | --- | --- | --- | --- | --- | --- | --- | --- | --- | --- |
| Models | | | | | | | P(M) | | P(M\|data) | | BF_M_ | | BF_10_ | | error % | |  |
| timepoint | | | | | |  | 0.200 |  | 0.613 |  | 6.343 |  | 1.000 |  |  |  |  |
| timepoint + Ordine | | | | | |  | 0.200 |  | 0.310 |  | 1.794 |  | 0.505 |  | 5.731 |  |  |
| timepoint + Ordine + timepoint ✻  Ordine | | | | | |  | 0.200 |  | 0.077 |  | 0.333 |  | 0.125 |  | 3.132 |  |  |
| Null model (incl. CRIq_tot, MRIq, subject, and random slopes) | | | | | |  | 0.200 |  | 2.657×10^-4^ |  | 0.001 |  | 4.332×10^-4^ |  | 1.991 |  |  |
| Ordine | | | | | |  | 0.200 |  | 1.138×10^-4^ |  | 4.551×10^-4^ |  | 1.855×10^-4^ |  | 3.262 |  |  |
|  | | | | | | | | | | | | | | | | |  |
| *Analysis of Effects* \| \| \| \| \| \| \| \| \| \| \|; Effects \| \| P(incl) \| \| P(excl) \| \| P(incl\|data) \| \| P(excl\|data) \| \| BF_incl_ \|; timepoint \| \| 0.600 \| \| 0.400 \| \| 1.000 \| \| 3.794×10^-4^ \| \| 1756.292 \|; Ordine \| \| 0.600 \| \| 0.400 \| \| 0.386 \| \| 0.614 \| \| 0.420 \|; timepoint ✻  Ordine \| \| 0.200 \| \| 0.800 \| \| 0.077 \| \| 0.923 \| \| 0.333 \| | | | | | | | | | | | | | | | | |  |
| *Post Hoc Comparisons - timepoint* | | | | | | | | | | | | | | | | |  |
|  | |  | | Prior Odds | | Posterior Odds | | | | BF_10, U_ | | | | error % | | | |
| 0 |  | 1 |  | 0.587 |  | 3.155 | | |  | 5.371 | | |  | 2.561×10^-7^ | | |  |
|  |  | 3 |  | 0.587 |  | 9.947 | | |  | 16.934 | | |  | 2.657×10^-7^ | | |  |
| 1 |  | 3 |  | 0.587 |  | 92.126 | | |  | 156.837 | | |  | 7.176×10^-8^ | | |  |
|  | | | | | | | | | | | | | | | | | |

| *Post Hoc Comparisons - Ordine* | | | | | | | | | | | |
| --- | --- | --- | --- | --- | --- | --- | --- | --- | --- | --- | --- |
|  | |  | | Prior Odds | | Posterior Odds | | BF_10, U_ | | error % | |
| C-M |  | M-C |  | 1.000 |  | 0.266 |  | 0.266 |  | 0.010 |  |
|  | | | | | | | | | | | |
|  | | | | | | | | | | | |

2.1.3. **RAVLT delayed recall**

| *Model Comparison* | | | | | | | | | | | |
| --- | --- | --- | --- | --- | --- | --- | --- | --- | --- | --- | --- |
| Models | | P(M) | | P(M\|data) | | BF_M_ | | BF_10_ | | error % | |
| timepoint |  | 0.200 |  | 0.568 |  | 5.251 |  | 1.000 |  |  |  |
| timepoint + Ordine |  | 0.200 |  | 0.346 |  | 2.121 |  | 0.610 |  | 4.201 |  |
| timepoint + Ordine + timepoint ✻  Ordine |  | 0.200 |  | 0.085 |  | 0.371 |  | 0.150 |  | 6.504 |  |
| Null model (incl. CRIq_tot, MRIq, subject, and random slopes) |  | 0.200 |  | 6.124×10^-4^ |  | 0.002 |  | 0.001 |  | 2.655 |  |
| Ordine |  | 0.200 |  | 3.220×10^-4^ |  | 0.001 |  | 5.673×10^-4^ |  | 3.221 |  |
|  | | | | | | | | | | | |
| *Analysis of Effects* \| \| \| \| \| \| \| \| \| \| \|; Effects \| \| P(incl) \| \| P(excl) \| \| P(incl\|data) \| \| P(excl\|data) \| \| BF_incl_ \|; timepoint \| \| 0.600 \| \| 0.400 \| \| 0.999 \| \| 9.344×10^-4^ \| \| 712.779 \|; Ordine \| \| 0.600 \| \| 0.400 \| \| 0.432 \| \| 0.568 \| \| 0.507 \|; timepoint ✻  Ordine \| \| 0.200 \| \| 0.800 \| \| 0.085 \| \| 0.915 \| \| 0.371 \| *Post Hoc Comparisons - timepoint* \| \| \| \| \| \| \| \| \| \| \|; \| \| \| \| Prior Odds \| \| Posterior Odds \| \| BF_10, U_ \| \| error % \|; 0 \| \| 1 \| \| 0.587 \| \| 2.190 \| \| 3.729 \| \| 3.848×10^-7^ \|; \| \| 3 \| \| 0.587 \| \| 5.205 \| \| 8.861 \| \| 2.324×10^-7^ \|; 1 \| \| 3 \| \| 0.587 \| \| 87.353 \| \| 148.712 \| \| 7.760×10^-8^ \| *Post Hoc Comparisons - Ordine* \| \| \| \| \| \| \| \| \| \| \|; \| \| \| \| Prior Odds \| \| Posterior Odds \| \| BF_10, U_ \| \| error % \|; C-M \| \| M-C \| \| 1.000 \| \| 0.336 \| \| 0.336 \| \| 0.010 \| 2.1.4. **Stroop test (time)** *Model Comparison* \| \| \| \| \| \| \| \| \| \| \|; Models \| \| P(M) \| \| P(M\|data) \| \| BF_M_ \| \| BF_10_ \| \| error % \|; timepoint \| \| 0.200 \| \| 0.558 \| \| 5.048 \| \| 1.000 \| \| \|; timepoint + Ordine \| \| 0.200 \| \| 0.347 \| \| 2.126 \| \| 0.622 \| \| 3.254 \|; timepoint + Ordine + timepoint ✻  Ordine \| \| 0.200 \| \| 0.088 \| \| 0.387 \| \| 0.158 \| \| 6.900 \|; Null model (incl. CRIq_tot, MRIq, subject, and random slopes) \| \| 0.200 \| \| 0.004 \| \| 0.018 \| \| 0.008 \| \| 2.002 \|; Ordine \| \| 0.200 \| \| 0.002 \| \| 0.010 \| \| 0.004 \| \| 2.712 \| *Analysis of Effects* \| \| \| \| \| \| \| \| \| \| \|; Effects \| \| P(incl) \| \| P(excl) \| \| P(incl\|data) \| \| P(excl\|data) \| \| BF_incl_ \|; timepoint \| \| 0.600 \| \| 0.400 \| \| 0.993 \| \| 0.007 \| \| 95.530 \|; Ordine \| \| 0.600 \| \| 0.400 \| \| 0.438 \| \| 0.562 \| \| 0.519 \|; timepoint ✻  Ordine \| \| 0.200 \| \| 0.800 \| \| 0.088 \| \| 0.912 \| \| 0.387 \| *Post Hoc Comparisons - timepoint* \| \| \| \| \| \| \| \| \| \| \|; \| \| \| \| Prior Odds \| \| Posterior Odds \| \| BF_10, U_ \| \| error % \|; 0 \| \| 1 \| \| 0.587 \| \| 0.194 \| \| 0.331 \| \| 0.019 \|; \| \| 3 \| \| 0.587 \| \| 61.143 \| \| 104.090 \| \| 1.351×10^-7^ \|; 1 \| \| 3 \| \| 0.587 \| \| 59.656 \| \| 101.559 \| \| 1.407×10^-7^ \| *Post Hoc Comparisons - Ordine* \| \| \| \| \| \| \| \| \| \| \|; \| \| \| \| Prior Odds \| \| Posterior Odds \| \| BF_10, U_ \| \| error % \|; C-M \| \| M-C \| \| 1.000 \| \| 0.600 \| \| 0.600 \| \| 0.009 \| 2.1.5. **SDMT** *Model Comparison* \| \| \| \| \| \| \| \| \| \| \|; Models \| \| P(M) \| \| P(M\|data) \| \| BF_M_ \| \| BF_10_ \| \| error % \|; timepoint \| \| 0.200 \| \| 0.513 \| \| 4.216 \| \| 1.000 \| \| \|; timepoint + Ordine \| \| 0.200 \| \| 0.373 \| \| 2.384 \| \| 0.728 \| \| 3.035 \|; timepoint + Ordine + timepoint ✻  Ordine \| \| 0.200 \| \| 0.100 \| \| 0.443 \| \| 0.194 \| \| 3.779 \|; Null model (incl. CRIq_tot, MRIq, subject, and random slopes) \| \| 0.200 \| \| 0.008 \| \| 0.033 \| \| 0.016 \| \| 1.162 \|; Ordine \| \| 0.200 \| \| 0.006 \| \| 0.022 \| \| 0.011 \| \| 2.515 \| | | | | | | | | | | | |

| *Analysis of Effects* | | | | | | | | | | | |
| --- | --- | --- | --- | --- | --- | --- | --- | --- | --- | --- | --- |
| Effects | | P(incl) | | P(excl) | | P(incl\|data) | | P(excl\|data) | | BF_incl_ | |
| timepoint |  | 0.600 |  | 0.400 |  | 0.986 |  | 0.014 |  | 48.185 |  |
| Ordine |  | 0.600 |  | 0.400 |  | 0.479 |  | 0.521 |  | 0.612 |  |
| timepoint ✻  Ordine |  | 0.200 |  | 0.800 |  | 0.100 |  | 0.900 |  | 0.443 |  |
|  | | | | | | | | | | | |

| *Post Hoc Comparisons - timepoint* | | | | | | | | | | | |
| --- | --- | --- | --- | --- | --- | --- | --- | --- | --- | --- | --- |
|  | |  | | *Prior Odds* | | *Posterior Odds* | | *BF_10, U_* | | *error %* | |
| 0 |  | *1* |  | *0.587* |  | *0.193* |  | *0.329* |  | *0.020* |  |
|  |  | *3* |  | *0.587* |  | *40.888* |  | *69.609* |  | *2.131×10^-7^* |  |
| 1 |  | *3* |  | *0.587* |  | *2.753* |  | *4.687* |  | *2.608×10^-7^* |  |
|  | | | | | | | | | | | |
|  | | | | | | | | | | | |

| *Post Hoc Comparisons - Ordine* | | | | | | | | | | | |
| --- | --- | --- | --- | --- | --- | --- | --- | --- | --- | --- | --- |
|  | |  | | Prior Odds | | Posterior Odds | | BF_10, U_ | | error % | |
| C-M |  | M-C |  | 1.000 |  | 0.378 |  | 0.378 |  | 0.010 |  |
|  | | | | | | | | | | | |
|  | | | | | | | | | | | |

2.1.6. **TMT-A**

| *Model Comparison* | | | | | | | | | | | | |
| --- | --- | --- | --- | --- | --- | --- | --- | --- | --- | --- | --- | --- |
| Models | | P(M) | | P(M\|data) | | BF_M_ | | | BF_10_ | | error % | |
| timepoint |  | 0.200 |  | 0.327 |  | 1.944 | |  | 1.000 |  |  |  |
| timepoint + Ordine |  | 0.200 |  | 0.200 |  | 1.002 | |  | 0.613 |  | 4.575 |  |
| Null model (incl. CRIq_tot, MRIq, subject, and random slopes) |  | 0.200 |  | 0.200 |  | 1.001 | |  | 0.612 |  | 3.754 |  |
| timepoint + Ordine + timepoint ✻  Ordine |  | 0.200 |  | 0.147 |  | 0.688 | |  | 0.449 |  | 4.929 |  |
| Ordine |  | 0.200 |  | 0.126 |  | 0.575 | |  | 0.384 |  | 4.301 |  |
|  | | | | | | | | | | | | |
| *Analysis of Effects* \| \| \| \| \| \| \| \| \| \| \|; Effects \| \| P(incl) \| \| P(excl) \| \| P(incl\|data) \| \| P(excl\|data) \| \| BF_incl_ \|; timepoint \| \| 0.600 \| \| 0.400 \| \| 0.674 \| \| 0.326 \| \| 1.379 \|; Ordine \| \| 0.600 \| \| 0.400 \| \| 0.473 \| \| 0.527 \| \| 0.598 \|; timepoint ✻  Ordine \| \| 0.200 \| \| 0.800 \| \| 0.147 \| \| 0.853 \| \| 0.688 \| *Post Hoc Comparisons - timepoint* \| \| \| \| \| \| \| \| \| \| \|; \| \| \| \| Prior Odds \| \| Posterior Odds \| \| BF_10, U_ \| \| error % \|; 0 \| \| 1 \| \| 0.587 \| \| 0.139 \| \| 0.237 \| \| 0.018 \|; \| \| 3 \| \| 0.587 \| \| 0.531 \| \| 0.905 \| \| 0.024 \|; 1 \| \| 3 \| \| 0.587 \| \| 5.476 \| \| 9.323 \| \| 2.276×10^-7^ \| *Post Hoc Comparisons - Ordine* \| \| \| \| \| \| \| \| \| \| \|; \| \| \| \| Prior Odds \| \| Posterior Odds \| \| BF_10, U_ \| \| error % \|; C-M \| \| M-C \| \| 1.000 \| \| 0.570 \| \| 0.570 \| \| 0.010 \| 2.1.7. **TMT-B** | | | | | | | | | | | | |
| Model Comparison | | | | | | | | | | | | |
| Models | | | P(M) | | P(M\|data) | | BF_M_ | | BF_10_ | | error % | |
| timepoint | |  | 0.200 |  | 0.435 |  | 3.077 |  | 1.000 |  |  |  |
| timepoint + Ordine | |  | 0.200 |  | 0.251 |  | 1.338 |  | 0.576 |  | 4.826 |  |
| Null model (incl. CRIq_tot, MRIq, subject, and random slopes) | |  | 0.200 |  | 0.174 |  | 0.840 |  | 0.399 |  | 3.875 |  |
| Ordine | |  | 0.200 |  | 0.090 |  | 0.395 |  | 0.207 |  | 5.157 |  |
| timepoint + Ordine + timepoint ✻  Ordine | |  | 0.200 |  | 0.051 |  | 0.216 |  | 0.118 |  | 5.287 |  |
|  | | | | | | | | | | | | |

| *Analysis of Effects* | | | | | | | | | | | |
| --- | --- | --- | --- | --- | --- | --- | --- | --- | --- | --- | --- |
| Effects | | P(incl) | | P(excl) | | P(incl\|data) | | P(excl\|data) | | BF_incl_ | |
| timepoint |  | 0.600 |  | 0.400 |  | 0.737 |  | 0.263 |  | 1.864 |  |
| Ordine |  | 0.600 |  | 0.400 |  | 0.392 |  | 0.608 |  | 0.429 |  |
| timepoint ✻  Ordine |  | 0.200 |  | 0.800 |  | 0.051 |  | 0.949 |  | 0.216 |  |
|  | | | | | | | | | | | |

| *Post Hoc Comparisons - timepoint* | | | | | | | | | | | |
| --- | --- | --- | --- | --- | --- | --- | --- | --- | --- | --- | --- |
|  | |  | | Prior Odds | | Posterior Odds | | BF_10, U_ | | error % | |
| 0 |  | 1 |  | 0.587 |  | 0.187 |  | 0.318 |  | 0.020 |  |
|  |  | 3 |  | 0.587 |  | 3.966 |  | 6.752 |  | 2.526×10^-7^ |  |
| 1 |  | 3 |  | 0.587 |  | 1.547 |  | 2.633 |  | 9.040×10^-7^ |  |
|  | | | | | | | | | | | |
|  | | | | | | | | | | | |

| *Post Hoc Comparisons - Ordine* | | | | | | | | | | | |
| --- | --- | --- | --- | --- | --- | --- | --- | --- | --- | --- | --- |
|  | |  | | Prior Odds | | Posterior Odds | | BF_10, U_ | | error % | |
| C-M |  | M-C |  | 1.000 |  | 0.307 |  | 0.307 |  | 0.010 |  |
|  | | | | | | | | | | | |
|  | | | | | | | | | | | |

2.1.8. **TMT-BA**

| *Model Comparison* | | | | | | | | | | | | |
| --- | --- | --- | --- | --- | --- | --- | --- | --- | --- | --- | --- | --- |
| Models | | P(M) | | P(M\|data) | | BF_M_ | | | BF_10_ | | error % | |
| Null model (incl. CRIq_tot, MRIq, subject, and random slopes) |  | 0.200 |  | 0.376 |  | 2.407 | |  | 1.000 |  |  |  |
| timepoint |  | 0.200 |  | 0.273 |  | 1.505 | |  | 0.728 |  | 2.539 |  |
| Ordine |  | 0.200 |  | 0.176 |  | 0.853 | |  | 0.468 |  | 3.050 |  |
| timepoint + Ordine |  | 0.200 |  | 0.142 |  | 0.662 | |  | 0.378 |  | 5.703 |  |
| timepoint + Ordine + timepoint ✻  Ordine |  | 0.200 |  | 0.033 |  | 0.138 | |  | 0.089 |  | 1.912 |  |
|  | | | | | | | | | | | | |
| *Analysis of Effects* \| \| \| \| \| \| \| \| \| \| \|; Effects \| \| P(incl) \| \| P(excl) \| \| P(incl\|data) \| \| P(excl\|data) \| \| BF_incl_ \|; timepoint \| \| 0.600 \| \| 0.400 \| \| 0.449 \| \| 0.551 \| \| 0.542 \|; Ordine \| \| 0.600 \| \| 0.400 \| \| 0.351 \| \| 0.649 \| \| 0.361 \|; timepoint ✻  Ordine \| \| 0.200 \| \| 0.800 \| \| 0.033 \| \| 0.967 \| \| 0.138 \| *Post Hoc Comparisons - timepoint* \| \| \| \| \| \| \| \| \| \| \|; \| \| \| \| Prior Odds \| \| Posterior Odds \| \| BF_10, U_ \| \| error % \|; 0 \| \| 1 \| \| 0.587 \| \| 0.181 \| \| 0.308 \| \| 0.020 \|; \| \| 3 \| \| 0.587 \| \| 0.406 \| \| 0.692 \| \| 0.024 \|; 1 \| \| 3 \| \| 0.587 \| \| 0.661 \| \| 1.126 \| \| 0.024 \| *Post Hoc Comparisons - Ordine* \| \| \| \| \| \| \| \| \| \| \|; \| \| \| \| Prior Odds \| \| Posterior Odds \| \| BF_10, U_ \| \| error % \|; C-M \| \| M-C \| \| 1.000 \| \| 0.264 \| \| 0.264 \| \| 0.010 \| | | | | | | |  |  |  |  |  |  |

2.2. **Well-being**
     2.2.1. **PANAS positive**

| *Model Comparison* | | | | | | | | | | | |
| --- | --- | --- | --- | --- | --- | --- | --- | --- | --- | --- | --- |
| Models | | P(M) | | P(M\|data) | | BF_M_ | | BF_10_ | | error % | |
| Null model (incl. MRIq, CRIq_tot, subject, and random slopes) |  | 0.200 |  | 0.516 |  | 4.272 |  | 1.000 |  |  |  |
| Ordine |  | 0.200 |  | 0.197 |  | 0.984 |  | 0.382 |  | 2.277 |  |
| timepoint |  | 0.200 |  | 0.195 |  | 0.971 |  | 0.378 |  | 1.643 |  |
| timepoint + Ordine |  | 0.200 |  | 0.072 |  | 0.308 |  | 0.139 |  | 2.025 |  |
| timepoint + Ordine + timepoint ✻  Ordine |  | 0.200 |  | 0.019 |  | 0.078 |  | 0.037 |  | 8.030 |  |
|  | | | | | | | | | | | |
| *Analysis of Effects* \| \| \| \| \| \| \| \| \| \| \|; Effects \| \| P(incl) \| \| P(excl) \| \| P(incl\|data) \| \| P(excl\|data) \| \| BF_incl_ \|; timepoint \| \| 0.600 \| \| 0.400 \| \| 0.286 \| \| 0.714 \| \| 0.267 \|; Ordine \| \| 0.600 \| \| 0.400 \| \| 0.288 \| \| 0.712 \| \| 0.270 \|; timepoint ✻  Ordine \| \| 0.200 \| \| 0.800 \| \| 0.019 \| \| 0.981 \| \| 0.078 \| | | | | | | | | | | | |

| *Post Hoc Comparisons - timepoint* | | | | | | | | | | | |
| --- | --- | --- | --- | --- | --- | --- | --- | --- | --- | --- | --- |
|  | |  | | Prior Odds | | Posterior Odds | | BF_10, U_ | | error % | |
| 0 |  | 1 |  | 0.587 |  | 0.153 |  | 0.260 |  | 0.019 |  |
|  |  | 3 |  | 0.587 |  | 0.192 |  | 0.326 |  | 0.020 |  |
| 1 |  | 3 |  | 0.587 |  | 0.413 |  | 0.704 |  | 0.024 |  |
|  | | | | | | | | | | | |

| *Post Hoc Comparisons - Ordine* | | | | | | | | | | | |
| --- | --- | --- | --- | --- | --- | --- | --- | --- | --- | --- | --- |
|  | |  | | Prior Odds | | Posterior Odds | | BF_10, U_ | | error % | |
| C-M |  | M-C |  | 1.000 |  | 0.367 |  | 0.367 |  | 0.010 |  |
|  | | | | | | | | | | | |

2.2.2. **PANAS negative**

| *Model Comparison* | | | | | | | | | | | |
| --- | --- | --- | --- | --- | --- | --- | --- | --- | --- | --- | --- |
| Models | | P(M) | | P(M\|data) | | BF_M_ | | BF_10_ | | error % | |
| timepoint |  | 0.200 |  | 0.350 |  | 2.155 |  | 1.000 |  |  |  |
| Null model (incl. CRIq_tot, MRIq, subject, and random slopes) |  | 0.200 |  | 0.242 |  | 1.279 |  | 0.692 |  | 2.995 |  |
| timepoint + Ordine |  | 0.200 |  | 0.203 |  | 1.018 |  | 0.580 |  | 4.765 |  |
| Ordine |  | 0.200 |  | 0.136 |  | 0.630 |  | 0.388 |  | 4.620 |  |
| timepoint + Ordine + timepoint ✻  Ordine |  | 0.200 |  | 0.069 |  | 0.295 |  | 0.196 |  | 4.656 |  |
|  | | | | | | | | | | | |
| *Analysis of Effects* \| \| \| \| \| \| \| \| \| \| \|; Effects \| \| P(incl) \| \| P(excl) \| \| P(incl\|data) \| \| P(excl\|data) \| \| BF_incl_ \|; timepoint \| \| 0.600 \| \| 0.400 \| \| 0.622 \| \| 0.378 \| \| 1.096 \|; Ordine \| \| 0.600 \| \| 0.400 \| \| 0.408 \| \| 0.592 \| \| 0.459 \|; timepoint ✻  Ordine \| \| 0.200 \| \| 0.800 \| \| 0.069 \| \| 0.931 \| \| 0.295 \| | | | | | | | | | | | |

| *Post Hoc Comparisons - timepoint* | | | | | | | | | | | |
| --- | --- | --- | --- | --- | --- | --- | --- | --- | --- | --- | --- |
|  | |  | | Prior Odds | | Posterior Odds | | BF_10, U_ | | error % | |
| 0 |  | 1 |  | 0.587 |  | 0.207 |  | 0.353 |  | 0.021 |  |
|  |  | 3 |  | 0.587 |  | 0.740 |  | 1.260 |  | 0.023 |  |
| 1 |  | 3 |  | 0.587 |  | 0.715 |  | 1.218 |  | 0.023 |  |
|  | | | | | | | | | | | |
| *Post Hoc Comparisons - Ordine* \| \| \| \| \| \| \| \| \| \| \|; \| \| \| \| Prior Odds \| \| Posterior Odds \| \| BF_10, U_ \| \| error % \|; C-M \| \| M-C \| \| 1.000 \| \| 0.756 \| \| 0.756 \| \| 0.010 \| | | | | | | | | | | | |

2.2.3. **SF-36 physical functioning**

| *Model Comparison* | | | | | | | | | | | | | | | | | | | | | | | |
| --- | --- | --- | --- | --- | --- | --- | --- | --- | --- | --- | --- | --- | --- | --- | --- | --- | --- | --- | --- | --- | --- | --- | --- |
| Models | | | | | | | P(M) | | P(M\|data) | | | | | | BF_M_ | | | BF_10_ | | | error % | | |
| Ordine | | | | | |  | 0.200 |  | 0.430 | | |  | | | 3.015 |  | | 1.000 | |  |  | |  |
| Null model (incl. CRIq_tot, MRIq, subject, and random slopes) | | | | | |  | 0.200 |  | 0.346 | | |  | | | 2.120 |  | | 0.806 | |  | 1.714 | |  |
| timepoint + Ordine | | | | | |  | 0.200 |  | 0.112 | | |  | | | 0.507 |  | | 0.262 | |  | 3.158 | |  |
| timepoint | | | | | |  | 0.200 |  | 0.084 | | |  | | | 0.369 |  | | 0.196 | |  | 3.477 | |  |
| timepoint + Ordine + timepoint ✻  Ordine | | | | | |  | 0.200 |  | 0.027 | | |  | | | 0.111 |  | | 0.063 | |  | 5.289 | |  |
|  | | | | | | | | | | | | | | | | | | | | | | | |
|  | | | | | | | | | | | | | | | | | | | | | | | |
| *Analysis of Effects* \| \| \| \| \| \| \| \| \| \| \|; Effects \| \| P(incl) \| \| P(excl) \| \| P(incl\|data) \| \| P(excl\|data) \| \| BF_incl_ \|; timepoint \| \| 0.600 \| \| 0.400 \| \| 0.224 \| \| 0.776 \| \| 0.192 \|; Ordine \| \| 0.600 \| \| 0.400 \| \| 0.569 \| \| 0.431 \| \| 0.881 \|; timepoint ✻  Ordine \| \| 0.200 \| \| 0.800 \| \| 0.027 \| \| 0.973 \| \| 0.111 \| Post Hoc Comparisons - timepoint | | | | | | | | | | | | | | | | | | | | | | | |
|  | |  | | Prior Odds | | Posterior Odds | | | | | | | | BF_10, U_ | | | | | | error % | | | |
| 0 |  | 1 |  | 0.587 |  | 0.226 | | | |  | | | | 0.385 | | | |  | | 0.021 | | |  |
|  |  | 3 |  | 0.587 |  | 0.271 | | | |  | | | | 0.461 | | | |  | | 0.022 | | |  |
| 1 |  | 3 |  | 0.587 |  | 0.148 | | | |  | | | | 0.252 | | | |  | | 0.018 | | |  |
|  | | | | | | | | | | | | | | | | | | | | | | | |
|  | | | | | | | | | | | | | | | | | | | | | | | |
|  | | | | | | | | | | | | | | | | | | | | | | | |
| Post Hoc Comparisons - Ordine | | | | | | | | | | | | | | | | | | | | | | | |
|  | |  | | Prior Odds | | Posterior Odds | | | | | | | BF_10, U_ | | | | | | error % | | | | |
| C-M |  | M-C |  | 1.000 |  | 0.262 | | | | |  | | 0.262 | | | |  | | 0.010 | | |  | |
|  | | | | | | | | | | | | | | | | | | | | | | | |
|  | | | | | | | | | | | | | | | | | | | | | | | |

2.2.4. **SF-36 role of limitations due to physical health**

| *Model Comparison* | | | | | | | | | | | | | | | | | | |
| --- | --- | --- | --- | --- | --- | --- | --- | --- | --- | --- | --- | --- | --- | --- | --- | --- | --- | --- |
| Models | | | | | | | P(M) | | P(M\|data) | | | | BF_M_ | | BF_10_ | | error % | |
| Null model (incl. CRIq_tot, MRIq, subject, and random slopes) | | | | | |  | 0.200 |  | 0.444 | |  | | 3.188 |  | 1.000 |  |  |  |
| Ordine | | | | | |  | 0.200 |  | 0.348 | |  | | 2.132 |  | 0.784 |  | 1.933 |  |
| timepoint + Ordine | | | | | |  | 0.200 |  | 0.075 | |  | | 0.325 |  | 0.169 |  | 3.489 |  |
| timepoint | | | | | |  | 0.200 |  | 0.071 | |  | | 0.308 |  | 0.161 |  | 4.526 |  |
| timepoint + Ordine + timepoint ✻  Ordine | | | | | |  | 0.200 |  | 0.062 | |  | | 0.266 |  | 0.140 |  | 2.071 |  |
|  | | | | | | | | | | | | | | | | | | |
|  | | | | | | | | | | | | | | | | | | |
|  | | | | | | | | | | | | | | | | | | |
| *Analysis of Effects* \| \| \| \| \| \| \| \| \| \| \|; Effects \| \| P(incl) \| \| P(excl) \| \| P(incl\|data) \| \| P(excl\|data) \| \| BF_incl_ \|; timepoint \| \| 0.600 \| \| 0.400 \| \| 0.209 \| \| 0.791 \| \| 0.176 \|; Ordine \| \| 0.600 \| \| 0.400 \| \| 0.485 \| \| 0.515 \| \| 0.628 \|; timepoint ✻  Ordine \| \| 0.200 \| \| 0.800 \| \| 0.062 \| \| 0.938 \| \| 0.266 \| Post Hoc Comparisons - timepoint | | | | | | | | | | | | | | | | | | |
|  | |  | | Prior Odds | | Posterior Odds | | | | | | BF_10, U_ | | | | error % | | |
| 0 |  | 1 |  | 0.587 |  | 0.232 | | | |  | | 0.395 | | |  | 0.022 | |  |
|  |  | 3 |  | 0.587 |  | 0.222 | | | |  | | 0.379 | | |  | 0.021 | |  |
| 1 |  | 3 |  | 0.587 |  | 0.137 | | | |  | | 0.234 | | |  | 0.018 | |  |
|  | | | | | | | | | | | | | | | | | | |

| *Post Hoc Comparisons - Ordine* | | | | | | | | | | | |
| --- | --- | --- | --- | --- | --- | --- | --- | --- | --- | --- | --- |
|  | |  | | Prior Odds | | Posterior Odds | | BF_10, U_ | | error % | |
| C-M |  | M-C |  | 1.000 |  | 0.568 |  | 0.568 |  | 0.010 |  |
|  | | | | | | | | | | | |

2.2.5. **SF-36 Role of limitations due to emotional problems**

| *Model Comparison* | | | | | | | | | | | |
| --- | --- | --- | --- | --- | --- | --- | --- | --- | --- | --- | --- |
| Models | | P(M) | | P(M\|data) | | BF_M_ | | BF_10_ | | error % | |
| Null model (incl. CRIq_tot, MRIq, subject, and random slopes) |  | 0.200 |  | 0.519 |  | 4.317 |  | 1.000 |  |  |  |
| Ordine |  | 0.200 |  | 0.323 |  | 1.910 |  | 0.623 |  | 17.127 |  |
| timepoint |  | 0.200 |  | 0.077 |  | 0.332 |  | 0.148 |  | 2.730 |  |
| timepoint + Ordine |  | 0.200 |  | 0.042 |  | 0.176 |  | 0.081 |  | 5.456 |  |
| timepoint + Ordine + timepoint ✻  Ordine |  | 0.200 |  | 0.039 |  | 0.161 |  | 0.075 |  | 3.372 |  |
|  | | | | | | | | | | | |
|  | | | | | | | | | | | |

| *Analysis of Effects* | | | | | | | | | | | |
| --- | --- | --- | --- | --- | --- | --- | --- | --- | --- | --- | --- |
| Effects | | P(incl) | | P(excl) | | P(incl\|data) | | P(excl\|data) | | BF_incl_ | |
| timepoint |  | 0.600 |  | 0.400 |  | 0.158 |  | 0.842 |  | 0.125 |  |
| Ordine |  | 0.600 |  | 0.400 |  | 0.404 |  | 0.596 |  | 0.452 |  |
| timepoint ✻  Ordine |  | 0.200 |  | 0.800 |  | 0.039 |  | 0.961 |  | 0.161 |  |
|  | | | | | | | | | | | |

| *Post Hoc Comparisons - timepoint* | | | | | | | | | | | |
| --- | --- | --- | --- | --- | --- | --- | --- | --- | --- | --- | --- |
|  | |  | | Prior Odds | | Posterior Odds | | BF_10, U_ | | error % | |
| 0 |  | 1 |  | 0.587 |  | 0.155 |  | 0.263 |  | 0.019 |  |
|  |  | 3 |  | 0.587 |  | 0.152 |  | 0.259 |  | 0.018 |  |
| 1 |  | 3 |  | 0.587 |  | 0.138 |  | 0.234 |  | 0.018 |  |
|  | | | | | | | | | | | |
|  | | | | | | | | | | | |

| *Post Hoc Comparisons - Ordine* | | | | | | | | | | | |
| --- | --- | --- | --- | --- | --- | --- | --- | --- | --- | --- | --- |
|  | |  | | Prior Odds | | Posterior Odds | | BF_10, U_ | | error % | |
| C-M |  | M-C |  | 1.000 |  | 2.030 |  | 2.030 |  | 0.009 |  |
|  | | | | | | | | | | | |
|  | | | | | | | | | | | |

2.2.6. **SF-36 Energy/Fatigue**

| *Model Comparison* | | | | | | | | | | | |
| --- | --- | --- | --- | --- | --- | --- | --- | --- | --- | --- | --- |
| Models | | P(M) | | P(M\|data) | | BF_M_ | | BF_10_ | | error % | |
| Null model (incl. CRIq_tot, MRIq, subject, and random slopes) |  | 0.200 |  | 0.414 |  | 2.821 |  | 1.000 |  |  |  |
| Ordine |  | 0.200 |  | 0.251 |  | 1.337 |  | 0.606 |  | 0.973 |  |
| timepoint |  | 0.200 |  | 0.185 |  | 0.910 |  | 0.448 |  | 1.124 |  |
| timepoint + Ordine |  | 0.200 |  | 0.120 |  | 0.544 |  | 0.289 |  | 2.395 |  |
| timepoint + Ordine + timepoint ✻  Ordine |  | 0.200 |  | 0.031 |  | 0.128 |  | 0.075 |  | 3.106 |  |
|  | | | | | | | | | | | |
|  | | | | | | | | | | | |

| *Analysis of Effects* | | | | | | | | | | | |
| --- | --- | --- | --- | --- | --- | --- | --- | --- | --- | --- | --- |
| Effects | | P(incl) | | P(excl) | | P(incl\|data) | | P(excl\|data) | | BF_incl_ | |
| timepoint |  | 0.600 |  | 0.400 |  | 0.336 |  | 0.664 |  | 0.337 |  |
| Ordine |  | 0.600 |  | 0.400 |  | 0.401 |  | 0.599 |  | 0.447 |  |
| timepoint ✻  Ordine |  | 0.200 |  | 0.800 |  | 0.031 |  | 0.969 |  | 0.128 |  |
|  | | | | | | | | | | | |

| *Post Hoc Comparisons - timepoint* | | | | | | | | | | | |
| --- | --- | --- | --- | --- | --- | --- | --- | --- | --- | --- | --- |
|  | |  | | Prior Odds | | Posterior Odds | | BF_10, U_ | | error % | |
| 0 |  | 1 |  | 0.587 |  | 0.137 |  | 0.233 |  | 0.018 |  |
|  |  | 3 |  | 0.587 |  | 0.423 |  | 0.720 |  | 0.024 |  |
| 1 |  | 3 |  | 0.587 |  | 0.342 |  | 0.581 |  | 0.023 |  |
|  | | | | | | | | | | | |
|  | | | | | | | | | | | |

| *Post Hoc Comparisons - Ordine* | | | | | | | | | | | |
| --- | --- | --- | --- | --- | --- | --- | --- | --- | --- | --- | --- |
|  | |  | | *Prior Odds* | | *Posterior Odds* | | *BF_10, U_* | | *error %* | |
| *C-M* |  | *M-C* |  | *1.000* |  | *0.502* |  | *0.502* |  | *0.010* |  |
|  | | | | | | | | | | | |
|  | | | | | | | | | | | |

2.2.7. **SF-36 Emotional wellbeing**

| *Model Comparison* | | | | | | | | | | | |
| --- | --- | --- | --- | --- | --- | --- | --- | --- | --- | --- | --- |
| Models | | P(M) | | P(M\|data) | | BF_M_ | | BF_10_ | | error % | |
| timepoint |  | 0.200 |  | 0.258 |  | 1.393 |  | 1.000 |  |  |  |
| timepoint + Ordine |  | 0.200 |  | 0.240 |  | 1.260 |  | 0.927 |  | 5.124 |  |
| Null model (incl. CRIq_tot, MRIq, subject, and random slopes) |  | 0.200 |  | 0.226 |  | 1.170 |  | 0.876 |  | 2.483 |  |
| Ordine |  | 0.200 |  | 0.198 |  | 0.990 |  | 0.768 |  | 3.779 |  |
| timepoint + Ordine + timepoint ✻  Ordine |  | 0.200 |  | 0.077 |  | 0.335 |  | 0.299 |  | 3.903 |  |
|  | | | | | | | | | | | |
|  | | | | | | | | | | | |

| *Analysis of Effects* | | | | | | | | | | | |
| --- | --- | --- | --- | --- | --- | --- | --- | --- | --- | --- | --- |
| Effects | | P(incl) | | P(excl) | | P(incl\|data) | | P(excl\|data) | | BF_incl_ | |
| timepoint |  | 0.600 |  | 0.400 |  | 0.575 |  | 0.425 |  | 0.903 |  |
| Ordine |  | 0.600 |  | 0.400 |  | 0.515 |  | 0.485 |  | 0.709 |  |
| timepoint ✻  Ordine |  | 0.200 |  | 0.800 |  | 0.077 |  | 0.923 |  | 0.335 |  |
|  | | | | | | | | | | | |

| *Post Hoc Comparisons - timepoint* | | | | | | | | | | | |
| --- | --- | --- | --- | --- | --- | --- | --- | --- | --- | --- | --- |
|  | |  | | Prior Odds | | Posterior Odds | | BF_10, U_ | | error % | |
| 0 |  | 1 |  | 0.587 |  | 0.182 |  | 0.310 |  | 0.020 |  |
|  |  | 3 |  | 0.587 |  | 0.775 |  | 1.319 |  | 0.023 |  |
| 1 |  | 3 |  | 0.587 |  | 0.364 |  | 0.620 |  | 0.023 |  |
|  | | | | | | | | | | | |

| *Post Hoc Comparisons - Ordine* | | | | | | | | | | | |
| --- | --- | --- | --- | --- | --- | --- | --- | --- | --- | --- | --- |
|  | |  | | Prior Odds | | Posterior Odds | | BF_10, U_ | | error % | |
| C-M |  | M-C |  | 1.000 |  | 3.577 |  | 3.577 |  | 0.008 |  |
|  | | | | | | | | | | | |
|  | | | | | | | | | | | |

2.2.8. **SF-36 Social functioning**

| *Model Comparison* | | | | | | | | | | | |
| --- | --- | --- | --- | --- | --- | --- | --- | --- | --- | --- | --- |
| Models | | P(M) | | P(M\|data) | | BF_M_ | | BF_10_ | | error % | |
| Null model (incl. CRIq_tot, MRIq, subject, and random slopes) |  | 0.200 |  | 0.440 |  | 3.138 |  | 1.000 |  |  |  |
| Ordine |  | 0.200 |  | 0.308 |  | 1.784 |  | 0.702 |  | 1.546 |  |
| timepoint |  | 0.200 |  | 0.118 |  | 0.535 |  | 0.268 |  | 1.233 |  |
| timepoint + Ordine |  | 0.200 |  | 0.082 |  | 0.356 |  | 0.186 |  | 1.353 |  |
| timepoint + Ordine + timepoint ✻  Ordine |  | 0.200 |  | 0.052 |  | 0.221 |  | 0.119 |  | 2.636 |  |
|  | | | | | | | | | | | |
|  | | | | | | | | | | | |

| *Analysis of Effects* | | | | | | | | | | | |
| --- | --- | --- | --- | --- | --- | --- | --- | --- | --- | --- | --- |
| Effects | | P(incl) | | P(excl) | | P(incl\|data) | | P(excl\|data) | | BF_incl_ | |
| timepoint |  | 0.600 |  | 0.400 |  | 0.252 |  | 0.748 |  | 0.225 |  |
| Ordine |  | 0.600 |  | 0.400 |  | 0.442 |  | 0.558 |  | 0.529 |  |
| timepoint ✻  Ordine |  | 0.200 |  | 0.800 |  | 0.052 |  | 0.948 |  | 0.221 |  |
|  | | | | | | | | | | | |

| *Post Hoc Comparisons - timepoint* | | | | | | | | | | | |
| --- | --- | --- | --- | --- | --- | --- | --- | --- | --- | --- | --- |
|  | |  | | Prior Odds | | Posterior Odds | | BF_10, U_ | | error % | |
| 0 |  | 1 |  | 0.587 |  | 0.151 |  | 0.257 |  | 0.018 |  |
|  |  | 3 |  | 0.587 |  | 0.184 |  | 0.314 |  | 0.020 |  |
| 1 |  | 3 |  | 0.587 |  | 0.292 |  | 0.496 |  | 0.023 |  |
|  | | | | | | | | | | | |
|  | | | | | | | | | | | |

| *Post Hoc Comparisons - Ordine* | | | | | | | | | | | |
| --- | --- | --- | --- | --- | --- | --- | --- | --- | --- | --- | --- |
|  | |  | | Prior Odds | | Posterior Odds | | BF_10, U_ | | error % | |
| C-M |  | M-C |  | 1.000 |  | 0.327 |  | 0.327 |  | 0.010 |  |
|  | | | | | | | | | | | |
|  | | | | | | | | | | | |

2.2.9. **SF-36 Pain**

| *Model Comparison* | | | | | | | | | | | |
| --- | --- | --- | --- | --- | --- | --- | --- | --- | --- | --- | --- |
| Models | | P(M) | | P(M\|data) | | BF_M_ | | BF_10_ | | error % | |
| Null model (incl. CRIq_tot, MRIq, subject, and random slopes) |  | 0.200 |  | 0.410 |  | 2.782 |  | 1.000 |  |  |  |
| Ordine |  | 0.200 |  | 0.364 |  | 2.294 |  | 0.889 |  | 1.025 |  |
| timepoint |  | 0.200 |  | 0.114 |  | 0.512 |  | 0.277 |  | 11.057 |  |
| timepoint + Ordine |  | 0.200 |  | 0.091 |  | 0.403 |  | 0.223 |  | 1.714 |  |
| timepoint + Ordine + timepoint ✻  Ordine |  | 0.200 |  | 0.020 |  | 0.083 |  | 0.049 |  | 3.785 |  |
|  | | | | | | | | | | | |
|  | | | | | | | | | | | |

| *Analysis of Effects* | | | | | | | | | | | |
| --- | --- | --- | --- | --- | --- | --- | --- | --- | --- | --- | --- |
| Effects | | P(incl) | | P(excl) | | P(incl\|data) | | P(excl\|data) | | BF_incl_ | |
| timepoint |  | 0.600 |  | 0.400 |  | 0.225 |  | 0.775 |  | 0.194 |  |
| Ordine |  | 0.600 |  | 0.400 |  | 0.476 |  | 0.524 |  | 0.606 |  |
| timepoint ✻  Ordine |  | 0.200 |  | 0.800 |  | 0.020 |  | 0.980 |  | 0.083 |  |
|  | | | | | | | | | | | |

| *Post Hoc Comparisons - timepoint* | | | | | | | | | | | |
| --- | --- | --- | --- | --- | --- | --- | --- | --- | --- | --- | --- |
|  | |  | | Prior Odds | | Posterior Odds | | BF_10, U_ | | error % | |
| 0 |  | 1 |  | 0.587 |  | 0.244 |  | 0.415 |  | 0.022 |  |
|  |  | 3 |  | 0.587 |  | 0.136 |  | 0.232 |  | 0.017 |  |
| 1 |  | 3 |  | 0.587 |  | 0.236 |  | 0.402 |  | 0.022 |  |
|  | | | | | | | | | | | |
|  | | | | | | | | | | | |

| *Post Hoc Comparisons - Ordine* | | | | | | | | | | | |
| --- | --- | --- | --- | --- | --- | --- | --- | --- | --- | --- | --- |
|  | |  | | Prior Odds | | Posterior Odds | | BF_10, U_ | | error % | |
| C-M |  | M-C |  | 1.000 |  | 0.442 |  | 0.442 |  | 0.010 |  |
|  | | | | | | | | | | | |
|  | | | | | | | | | | | |

2.2.10. **SF-36 General Health**

| *Model Comparison* | | | | | | | | | | | | | | | | |
| --- | --- | --- | --- | --- | --- | --- | --- | --- | --- | --- | --- | --- | --- | --- | --- | --- |
| Models | | | | | | | P(M) | | P(M\|data) | | BF_M_ | | BF_10_ | | error % | |
| Null model (incl. CRIq_tot, MRIq, subject, and random slopes) | | | | | |  | 0.200 |  | 0.412 |  | 2.805 |  | 1.000 |  |  |  |
| timepoint | | | | | |  | 0.200 |  | 0.225 |  | 1.164 |  | 0.547 |  | 0.766 |  |
| Ordine | | | | | |  | 0.200 |  | 0.215 |  | 1.098 |  | 0.522 |  | 0.897 |  |
| timepoint + Ordine | | | | | |  | 0.200 |  | 0.119 |  | 0.538 |  | 0.288 |  | 1.013 |  |
| timepoint + Ordine + timepoint ✻  Ordine | | | | | |  | 0.200 |  | 0.029 |  | 0.118 |  | 0.069 |  | 8.599 |  |
|  | | | | | | | | | | | | | | | | |
|  | | | | | | | | | | | | | | | | |
| *Analysis of Effects* | | | | | | | | | | | | | | | | |
| Effects | | P(incl) | | P(excl) | | P(incl\|data) | | P(excl\|data) | | BF_incl_ | |  |  |  |  |  |
| timepoint |  | 0.600 |  | 0.400 |  | 0.372 |  | 0.628 |  | 0.396 |  |  |  |  |  |  |
| Ordine |  | 0.600 |  | 0.400 |  | 0.362 |  | 0.638 |  | 0.379 |  |  |  |  |  |  |
| timepoint ✻  Ordine |  | 0.200 |  | 0.800 |  | 0.029 |  | 0.971 |  | 0.118 |  |  |  |  |  |  |
|  | | | | | | | | | | | |  |  |  |  |  |

| *Post Hoc Comparisons - timepoint* | | | | | | | | | | | |
| --- | --- | --- | --- | --- | --- | --- | --- | --- | --- | --- | --- |
|  | |  | | Prior Odds | | Posterior Odds | | BF_10, U_ | | error % | |
| 0 |  | 1 |  | 0.587 |  | 0.468 |  | 0.797 |  | 0.024 |  |
|  |  | 3 |  | 0.587 |  | 0.137 |  | 0.233 |  | 0.018 |  |
| 1 |  | 3 |  | 0.587 |  | 0.511 |  | 0.870 |  | 0.024 |  |
|  | | | | | | | | | | | |

| *Post Hoc Comparisons - Ordine* | | | | | | | | | | | |
| --- | --- | --- | --- | --- | --- | --- | --- | --- | --- | --- | --- |
|  | |  | | Prior Odds | | Posterior Odds | | BF_10, U_ | | error % | |
| C-M |  | M-C |  | 1.000 |  | 0.459 |  | 0.459 |  | 0.010 |  |
|  | | | | | | | | | | | |
|  | | | | | | | | | | | |

2.2.11. **SF-36 Health change**

| *Model Comparison* | | | | | | | | | | | | | | | | |
| --- | --- | --- | --- | --- | --- | --- | --- | --- | --- | --- | --- | --- | --- | --- | --- | --- |
| Models | | | | | | | P(M) | | P(M\|data) | | BF_M_ | | BF_10_ | | error % | |
| Null model (incl. CRIq_tot, MRIq, subject, and random slopes) | | | | | |  | 0.200 |  | 0.539 |  | 4.680 |  | 1.000 |  |  |  |
| Ordine | | | | | |  | 0.200 |  | 0.232 |  | 1.211 |  | 0.431 |  | 1.846 |  |
| timepoint + Ordine | | | | | |  | 0.200 |  | 0.138 |  | 0.640 |  | 0.256 |  | 76.419 |  |
| timepoint | | | | | |  | 0.200 |  | 0.074 |  | 0.322 |  | 0.138 |  | 1.530 |  |
| timepoint + Ordine + timepoint ✻  Ordine | | | | | |  | 0.200 |  | 0.016 |  | 0.065 |  | 0.030 |  | 3.064 |  |
|  | | | | | | | | | | | | | | | | |
|  | | | | | | | | | | | | | | | | |
| *Analysis of Effects* | | | | | | | | | | | | | | | | |
| Effects | | P(incl) | | P(excl) | | P(incl\|data) | | P(excl\|data) | | BF_incl_ | |  |  |  |  |  |
| timepoint |  | 0.600 |  | 0.400 |  | 0.228 |  | 0.772 |  | 0.197 |  |  |  |  |  |  |
| Ordine |  | 0.600 |  | 0.400 |  | 0.386 |  | 0.614 |  | 0.420 |  |  |  |  |  |  |
| timepoint ✻  Ordine |  | 0.200 |  | 0.800 |  | 0.016 |  | 0.984 |  | 0.065 |  |  |  |  |  |  |
|  | | | | | | | | | | | |  |  |  |  |  |

| *Post Hoc Comparisons - timepoint* | | | | | | | | | | | |
| --- | --- | --- | --- | --- | --- | --- | --- | --- | --- | --- | --- |
|  | |  | | Prior Odds | | Posterior Odds | | BF_10, U_ | | error % | |
| 0 |  | 1 |  | 0.587 |  | 0.149 |  | 0.253 |  | 0.018 |  |
|  |  | 3 |  | 0.587 |  | 0.136 |  | 0.232 |  | 0.017 |  |
| 1 |  | 3 |  | 0.587 |  | 0.140 |  | 0.239 |  | 0.018 |  |
|  | | | | | | | | | | | |
|  | | | | | | | | | | | |

| *Post Hoc Comparisons - Ordine* | | | | | | | | | | | |
| --- | --- | --- | --- | --- | --- | --- | --- | --- | --- | --- | --- |
|  | |  | | *Prior Odds* | | *Posterior Odds* | | *BF_10, U_* | | *error %* | |
| *C-M* |  | *M-C* |  | *1.000* |  | *0.286* |  | *0.286* |  | *0.010* |  |
|  | | | | | | | | | | | |
|  | | | | | | | | | | | |
